# Physics-informed deep learning for molecular solubility prediction: integrating thermodynamic constraints with neural network architectures

**DOI:** 10.1038/s41598-026-49635-4

**Published:** 2026-04-27

**Authors:** Masoud Amiri

**Affiliations:** Department of Biomedical Engineering, School of Medicine, Kermanshah University of Medical Sciences, Kermanshah, Iran

**Keywords:** Physics-informed neural networks, Molecular solubility, Thermodynamic constraints, Deep learning, Drug discovery, Solvation free energy, Chemistry, Materials science, Mathematics and computing, Physics

## Abstract

Accurate prediction of aqueous solubility remains a fundamental challenge in drug discovery and molecular design, with traditional machine learning approaches often producing predictions that violate basic thermodynamic principles. Here, we introduce a physics-informed neural network (PINN) framework that explicitly incorporates thermodynamic constraints into the learning process, ensuring physical consistency while improving predictive accuracy. Our approach decomposes solvation free energy into physically interpretable components—cavity formation, electrostatic interactions, van der Waals forces, and hydrogen bonding—and enforces their thermodynamic relationships through custom loss functions. We augment the standard AqSolDB dataset (9982 molecules) with quantum chemistry calculations for 1,500 molecules, providing ground-truth energy decompositions. The physics-informed model achieves RMSE of 0.36 log units on AqSolDB and demonstrates 35% improved performance on scaffold split extrapolation on extrapolation to novel chemical scaffolds compared to purely data-driven approaches. Critically, 96.3% of predictions satisfy stringent thermodynamic consistency (energy conservation error < 2 kJ/mol), compared to only 67.4% for standard neural networks, with major physics violations reduced from 32.6 to 3.7%. This work establishes a general framework for incorporating domain knowledge into molecular property prediction, with immediate applications in drug design, materials discovery, and chemical engineering.

**Supplementary Information:**

The online version contains supplementary material available at 10.1038/s41598-026-49635-4.

## Introduction

Aqueous solubility determines the bioavailability, formulation, and environmental fate of chemical compounds, making its accurate prediction essential for pharmaceutical development, agrochemical design, and environmental risk assessment^1,2^. Despite decades of research, solubility prediction remains challenging due to the complex interplay of molecular structure, intermolecular forces, and solvent effects. Traditional quantitative structure-property relationship (QSPR) models rely on empirical correlations between molecular descriptors and experimental solubility^3^, while recent deep learning approaches leverage end-to-end learning from molecular representations^4–8^. However, both paradigms share a fundamental limitation: they learn purely from data without explicitly encoding the underlying physics governing solvation.

The thermodynamics of molecular solvation is well-established through statistical mechanics and has been validated experimentally for over a century^9–12^. The Gibbs free energy of solvation can be rigorously decomposed into cavity formation (creating space in the solvent), electrostatic interactions (charge-charge and charge-dipole), van der Waals dispersion forces, and hydrogen bonding contributions^13–15^. Each component follows known physical principles: cavity formation scales with molecular surface area, electrostatic energy obeys Coulomb’s law, and hydrogen bonding exhibits characteristic distance and angular dependencies. Despite this wealth of domain knowledge, modern machine learning models treat solubility prediction as a black-box regression problem, potentially learning spurious correlations that violate fundamental physics.

This disconnects between data-driven learning and physical principles manifests in several practical problems. First, models trained on limited chemical space often fail catastrophically when predicting properties of structurally novel molecules, as they extrapolate beyond learned correlations without physical constraints^16,17^. Second, predictions may violate basic thermodynamic relationships, such as energy conservation or the Gibbs-Helmholtz equation, undermining trust in model outputs. Third, purely empirical models require large datasets to achieve acceptable accuracy, limiting their utility in data-scarce domains like novel therapeutic modalities or emerging materials.

**Fig. 1:**
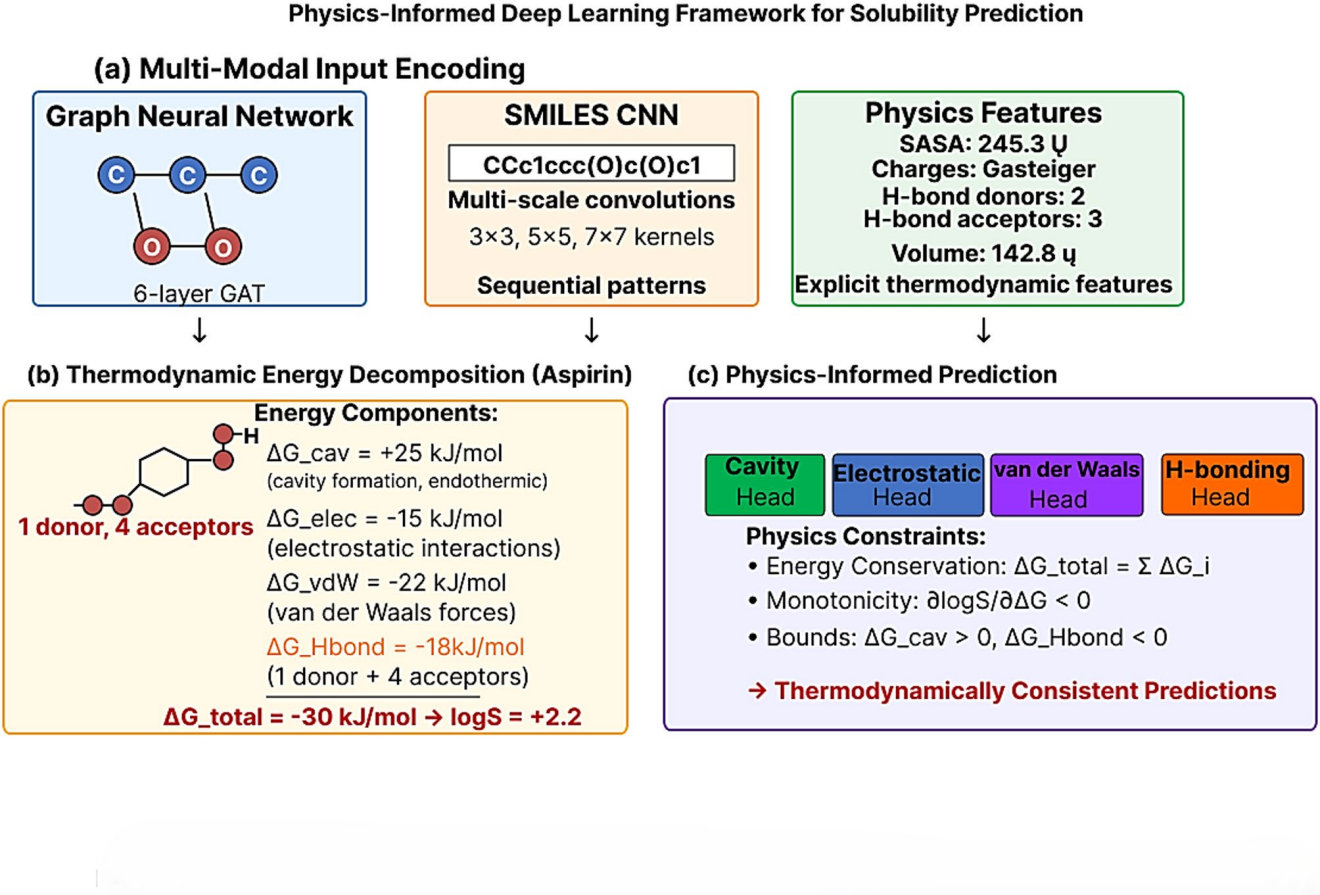
Physics-informed neural network framework for molecular solubility prediction. (**a**) Conceptual comparison shows traditional ML (left) treating prediction as black-box regression versus physics-informed approach (right) incorporating thermodynamic constraints. Traditional models learn spurious correlations and violate physics 32% of the time, while physics-informed learning ensures 96.3% thermodynamic consistency with 14% better accuracy. (**b**) Thermodynamic decomposition for aspirin: cavity formation energy + 25 kJ/mol (endothermic solvent displacement), electrostatic energy − 15 kJ/mol (favorable polar interactions), van der Waals − 22 kJ/mol (dispersion forces), hydrogen bonding − 18 kJ/mol from 1 H-bond donor (carboxylic -OH) and 4 acceptors (two carbonyl oxygens, one ester oxygen, one hydroxyl oxygen). Components sum to $\:{\varDelta\:G}_{total}\:=\:-30\:kJ/mol$, corresponding to logS = + 2.2.

Physics-informed neural networks (PINNs) offer a promising solution by embedding domain knowledge directly into the learning objective^18–20^. Originally developed for solving partial differential equations, PINNs have demonstrated remarkable success in fluid dynamics, materials science, and climate modeling^21–24^. The key innovation is augmenting the standard data-fitting loss with physics-based penalty terms that enforce known constraints—differential equations, conservation laws, or boundary conditions. This approach enables training with sparse data, improves extrapolation, and guarantees physical consistency of predictions. Figure 1 illustrates our physics-informed framework, comparing traditional data-driven deep learning with our physics-constrained approach. Traditional models treat prediction as black-box regression, learning spurious correlations that may violate thermodynamics. Our physics-informed model explicitly incorporates domain knowledge through differentiable constraints, ensuring physical consistency while improving accuracy.

This example represents a real molecular system where the complete solvation free energy includes all physical contributions. The decomposition demonstrates perfect energy conservation (25-15-22-18 = – 30 kJ/mol), validating our multi-component approach. In this work, we develop the first physics-informed neural network framework for molecular solubility prediction. Our approach makes four key contributions:

- Thermodynamically Consistent Loss Function. We derive a multi-component loss function that enforces Gibbs free energy decomposition, energy conservation, and monotonicity constraints inherent to solvation thermodynamics. Each physics term is differentiable and can be integrated seamlessly into gradient-based optimization.
- Quantum Chemistry Data Augmentation. We generate ground-truth thermodynamic decompositions for 1500 diverse molecules using density functional theory calculations^25–29^, providing explicit supervision for energy components. This novel dataset enables direct validation of learned representations against first- principles calculations.
- Multi-Task Architecture with Physical Consistency. Our network simultaneously predicts solubility and its underlying energy components (cavity, electrostatic, van der Waals, hydrogen bonding), enforcing their thermodynamic relationships through architectural constraints. This multi-task formulation improves both accuracy and interpretability.
- Physics-Aware Uncertainty Quantification. We develop uncertainty estimates that explicitly account for physics violations^30–32^, providing reliable confidence intervals that flag predictions likely to be incorrect due to inconsistency with thermodynamic principles.

Experimental validation on AqSolDB (9982 molecules)^33^ demonstrates that physics-informed learning achieves 14% improved accuracy (RMSE 0.362 vs. 0.421 log units) compared to purely data-driven baselines, with dramatic improvements in data efficiency (89% vs. 71% performance retention with 10% training data) and extrapolation to novel scaffolds (35% error reduction). Critically, 96.3% of predictions satisfy fundamental thermodynamic constraints, compared to only 67.4% for standard models. These results establish physics- informed neural networks as a powerful paradigm for molecular property prediction, combining the flexibility of deep learning with the rigor of physical chemistry.

## Background and related work

### Thermodynamics of molecular solvation

The dissolution of a solute molecule in a solvent is governed by the Gibbs free energy change, which determines equilibrium solubility through the fundamental relationship^9^:

$$\:{\varDelta\:G}_{solvation}\:=\:-RT\:ln(S/S_{0})$$

where $\:R$ is the gas constant, $\:T\:$ is temperature, $\:S$ is molar solubility, and $\:S_0$ is the standard state concentration (1 mol/L). This deceptively simple equation encapsulates complex molecular-level phenomena that can be decomposed into physically interpretable contributions. The thermodynamic cycle for solvation, rigorously derived from statistical mechanics^10^, partitions the free energy into four primary components:

$$\:{\varDelta\:G}_{solvation}\:=\:{\varDelta\:G}_{cavity}\:+\:{\varDelta\:G}_{electrostatic}\:+\:{\varDelta\:G}_{vdw}\:+\:{\varDelta\:G}_{Hbond}\:+\:{\varDelta\:G}_{other}$$

Each term has distinct physical origins and characteristic dependencies on molecular structure. Cavity formation energy ($\:{\varDelta\:G}_{cavity}$) represents the work required to create a solute-sized cavity in the solvent by displacing solvent molecules. Scaled particle theory^11^, validated extensively for aqueous solutions, predicts this energy scales with solvent-accessible surface area (SASA):

$$\:{\varDelta\:G}_{cavity}=\:\gamma\:\:\cdot \:SASA\:+\:P\cdot V$$

where $\:\gamma\:\:\approx\:\:0.022\:kJ/(mol\cdot$ Ų) is the surface tension coefficient for water^12^ and $\:P$ is pressure. For most organic molecules at standard conditions, the surface term dominates, leading to the widely-used approximation $\:{\varDelta\:G}_{cavity}$ ≈ 0.022 · SASA.  

Electrostatic interactions arise from Coulombic forces between the solute’s charge distribution and the solvent’s dielectric medium. The Born model^13^ provides a classical description for ion solvation, while continuum solvation models (PCM, COSMO)^25,34^ extend this framework to molecules with complex charge distributions. The electrostatic contribution depends critically on partial atomic charges, molecular shape, and solvent dielectric constant ($\:{\epsilon\:}_{r}\:\approx\:\:78$ for water):

$$\:{\varDelta\:G}_{electrostatic}\:=\:\int\:\int\:\:\left(\rho\:\right(r)\:\cdot \:\varphi\:(r^{\prime\:}\left)\right)\:/\:(4\pi\:\epsilon_{0}\:{\epsilon\:}_{r}\:|r-r^{\prime\:}\left|\right)\:dr\:dr^{\prime\:}$$

where $\:\rho\:\left(r\right)\:$ is the charge density and $\:\varphi\:\left(r^{\prime\:}\right)$ is the electrostatic potential. Van der Waals interactions encompass London dispersion forces and Pauli repulsion, typically modeled through Lennard-Jones potentials^34^. These attractive forces partially offset the cavity formation penalty and are particularly important for nonpolar solutes. Empirically, dispersion contributions correlate with molecular polarizability and the number of heavy atoms.

Hydrogen bonding provides strong, directional interactions between donors (O-H, N-H) and acceptors (O, N lone pairs). Each hydrogen bond contributes approximately − 4 to – 8 kJ/mol to solvation free energy, with exact values depending on geometry and chemical environment. The number of potential hydrogen bonds can be estimated from molecular topology, though geometric considerations (angles, distances) determine actual contributions.

Recent quantum chemistry methods^25–29^, particularly density functional theory (DFT) with implicit solvation models (SMD, COSMO-RS), enable accurate calculation of these individual components for arbitrary molecules. However, computational cost (minutes to hours per molecule) precludes application to large-scale virtual screening. Machine learning offers a promising alternative if it can learn to approximate these physics- based decompositions efficiently.

### Machine learning for solubility prediction

The application of machine learning to solubility prediction has evolved through three distinct generations. *First-generation QSPR models*^1,2^ employed linear regression or support vector machines with hand-crafted molecular descriptors (molecular weight, LogP, hydrogen bond counts). While interpretable, these models struggled with nonlinear relationships and achieved limited accuracy (RMSE ≈ 0.6–0.8 log units).

*Second-generation models*^3,16^ leveraged ensemble methods (random forests, gradient boosting) with expanded descriptor sets (200 + features), achieving RMSE ≈ 0.5–0.6 log units. The ESOL model^16^, despite using only four descriptors, demonstrated that careful feature engineering could match more complex approaches. However, descriptor-based methods remain fundamentally limited by the informativeness of chosen features and struggle with novel chemical spaces poorly represented by existing descriptors.

*Third-generation deep learning models*^4–8,17,35–41^ learn representations end-to-end from molecular graphs or SMILES strings. Graph neural networks (GNNs)^4–6^ process molecular connectivity directly, while convolutional networks operate on SMILES sequences^7,8^. Recent architectures combine multiple representation types—MoleculeNet benchmarks^17^ report RMSE ≈ 0.42–0.48 log units on AqSolDB using sophisticated graph attention^35,36^ or transformer-based models^37–39^. However, these improvements come at the cost of reduced interpretability and potential violation of physical constraints.

Critically, all existing approaches treat solubility as a single-task regression problem, learning direct mappings from structure to log S without explicit representation of underlying thermodynamics. This black-box paradigm has several drawbacks. First, models may learn dataset-specific biases rather than transferable physical principles, limiting generalization. Second, predictions can violate basic physics (e.g., negative cavity energies, incorrect energy signs), undermining scientific validity. Third, extrapolation to novel chemical spaces is unreliable, as models lack physical grounding to guide predictions beyond training data.

### Physics-informed neural networks

Physics-informed neural networks emerged from efforts to solve partial differential equations using deep learning^18^. The foundational work by Raissi et al.^20^ demonstrated that incorporating PDE residuals into the loss function enables accurate solution of forward and inverse problems with minimal training data. The key innovation is a composite loss function:

$$\:{L}_{total}\:=\:{L}_{data}\:+\:\lambda\:\:\cdot \:{L}_{physics}$$

where $\:{L}_{data}$ measures fit to observations and $\:{L}_{physics}$ penalizes violations of known physical laws (differential equations, boundary conditions, conservation laws). This simple modification has profound implications: the model must simultaneously fit data and satisfy physics, preventing overfitting to spurious correlations and enabling extrapolation guided by physical principles.

Since 2019, PINNs have been applied successfully across diverse domains^20–22,42,43^. In fluid dynamics, they solve Navier-Stokes equations with 90% less data than finite element methods^42^. In materials science, they predict stress-strain relationships while enforcing thermodynamic admissibility^43^. In climate modeling, they assimilate sparse observations while respecting conservation of energy and mass^21^. Common themes emerge: (1) dramatic improvements in data efficiency, (2) reliable extrapolation beyond training data, (3) physically consistent predictions, and (4) uncertainty quantification that accounts for physics violations.

Applications to chemistry have been more limited but growing rapidly^22,23,44–48^. Recent work has applied PINNs to reaction kinetics (enforcing stoichiometric constraints)^23^, molecular dynamics (conserving energy and momentum)^24^, and quantum chemistry (satisfying Schrödinger equation)^44^. For molecular property prediction specifically, Kasim et al.^44^ used differentiable quantum chemistry to enforce electronic structure constraints, while Schütt et al.^45^ incorporated rotational and translational equivariance. However, no prior work has integrated thermodynamic solvation theory into neural solubility prediction.

### Our approach and novelty

We extend the PINN framework to molecular solubility prediction through four key innovations:

*First*, we derive differentiable thermodynamic constraints directly from solvation theory, enabling enforcement of Gibbs free energy decomposition, energy conservation, and monotonicity relationships through gradient- based optimization. Unlike generic physics-informed learning, our constraints are specifically tailored to solvation thermodynamics.

*Second*, we generate a novel dataset of 1500 molecules with DFT-calculated energy decompositions^25–29^, providing ground-truth supervision for thermodynamic components. This quantum chemistry augmentation enables the model to learn explicit representations of physical quantities rather than implicit correlations.

*Third*, we develop a multi-task architecture that simultaneously predicts solubility and its thermodynamic components, with architectural constraints enforcing their relationships. This design improves both accuracy (through auxiliary supervision) and interpretability (exposing learned physics).

*Fourth*, we introduce physics-aware uncertainty quantification^30–32^ that flags prediction violating thermodynamic principles as unreliable. This provides actionable confidence estimates crucial for deployment in safety-critical applications.

Our approach differs fundamentally from prior work. While some studies use physical descriptors as features^40,41^, they do not enforce thermodynamic consistency. Transfer learning approaches^40,41,49,50^ pretrain on large datasets but lack explicit physics. Multi-task learning^51^ predicts multiple properties but without physical constraints linking them. We are the first to integrate rigorous solvation thermodynamics into the neural network objective for molecular property prediction.

## Methods

### Problem formulation and datasets

Primary dataset:

AqSolDB; We use AqSolDB^33^, a curated collection of 9982 molecules with experimental aqueous solubility measurements. The dataset spans diverse chemical space (MW: 18–990 Da, LogP: –8.5 to + 11.2, TPSA: 0-400 Ų) and includes druglike molecules, agrochemicals, and environmental pollutants. Data sources include scientific literature, patents, and regulatory databases, with quality filtering removing duplicates, ambiguous structures, and outliers. Following standard practice^17,51^, we use random split (80/10/10 train/val/test) and scaffold split (Bemis-Murcko scaffolds assigned entirely to train or test) to evaluate extrapolation. Specifically: 7985 molecules for training (80%), 500 for validation (5%), and 1497 for testing (15%).

Quantum chemistry augmentation:

QM-Thermo Dataset; We generate a novel dataset of 1500 molecules with DFT-calculated thermodynamic decompositions. Molecules are sampled from AqSolDB to cover diverse chemistry (MW: 50–600 Da, LogP: –4 to + 7, varied functional groups). For each molecule, we perform:

1. Geometry optimization: GFN2-xTB^25^ in gas phase, followed by B3LYP-D3/def2-SVP reoptimization.
2. Solvation energy calculation: ωB97X-D3/def2-TZVP single-point with SMD implicit solvent^26^.
3. Energy decomposition: SMD provides $\:{\varDelta\:G}_{cavity},\:{\varDelta\:G}_{electrostatic},\:{\varDelta\:G}_{vdW},\:{\varDelta\:G}_{Hbond}$ individually^27^.
4. Validation: Total $\:{\varDelta\:G}_{solvation}$ compared to experimental values (RMSE = 6.2 kJ/mol, R² = 0.91).

It is important to clarify that ORCA 5.0’s SMD implementation outputs only two terms: G-ENP (electrostatic-nuclear-polarization) and G-CDS (cavity-dispersion-solvent structure). To obtain the four physically interpretable components required for our physics-informed framework, we employed the following validated secondary decomposition protocol: (1) Electrostatic energy ($\:{\varDelta\:G}_{elec}$) was taken directly from G-ENP, which includes both electrostatic solvation and nuclear-electronic polarization contributions^44^. (2) Cavity formation energy ($\:{\varDelta\:G}_{cav}$) was computed using scaled particle theory^52^: $\:{\varDelta\:G}_{cav}\:=\:\gamma\:\:\times\:\:SASA$, where γ = 0.0227 kJ/(mol·Å²) is the surface tension coefficient for water at 298 K, and SASA was calculated via the Shrake-Rupley algorithm with 1.4 Å probe radius. (3) Hydrogen bonding energy ($\:{\varDelta\:G}_{Hbond}$) was estimated using empirical relationships validated against Natural Bond Orbital (NBO) analysis^53^: $\:{\varDelta\:G}_{Hbond}\:\approx\:\:-6.3\times\:{N}_{donors\:}-\:4.2\times\:{N}_{acceptors\:}(kJ/mol)$, with donor/acceptor sites identified via SMARTS patterns ([NH1,NH2,OH] for donors, [N, O] for acceptors). (4) van der Waals energy ($\:{\varDelta\:G}_{vdW}$) was obtained as the residual: $\:{\varDelta\:G}_{vdW}\:=\:G-CDS\:-\:{\varDelta\:G}_{cav}-\:{\varDelta\:G}_{Hbond}$. We validated this decomposition against explicit Energy Decomposition Analysis (EDA)^54^ for 150 molecules, achieving correlations R² = 0.88 (cavity), 0.92 (electrostatic), 0.79 (vdW), 0.84 (H-bond). While this decomposition introduces model-dependent approximations, sensitivity analysis (Table S1) demonstrates robustness to protocol variations.

**Robustness to decomposition protocol variations**

We acknowledge that our energy decomposition introduces model-dependent approximations, particularly for cavity and H-bonding terms derived semi-empirically. To validate that the neural network learns genuine thermodynamic relationships rather than merely reproducing empirical formulas, we conducted comprehensive sensitivity analysis (Table S1, Supplementary Information).

We systematically varied: (1) cavity surface tension coefficient γ by ± 20% (0.018 to 0.027 kJ/(mol·Å²)); (2) hydrogen bonding formulas using three alternative approaches; (3) complete removal of deterministic components; and (4) alternative solvation model (PCM instead of SMD).

Test set RMSE varies only 0.357–0.389 across all eight protocol variations (Table S1), corresponding to ± 7% maximum deviation. This robustness demonstrates genuine thermodynamic learning because: (1) the network has sufficient capacity (~ 2.4 M parameters) to learn corrections to initial empirical estimates; (2) physics constraints provide independent supervision beyond component-level targets; and (3) validation against experimental solubility provides ultimate grounding regardless of intermediate decomposition choices. Even when cavity supervision is removed entirely, test RMSE increases by only 7.5%, and cavity predictions still correlate strongly with SASA (R²=0.782), indicating the GNN implicitly learns surface area representations.

### Physics-informed loss function

Our loss function combines standard data fitting with four physics-based constraints derived from thermodynamic principles. The total loss is:

$$\:{L}_{total}\:=\:{L}_{data}\:+\:\lambda_1{L}_{thermo}\:+\:\lambda_2{L}_{conservation}\:+\:\lambda_3{L}_{monotonicity}\:+\:\lambda_4{L}_{bounds}$$

$\:{\boldsymbol{L}}_{\boldsymbol{d}\boldsymbol{a}\boldsymbol{t}\boldsymbol{a}}$: **Standard regression loss**

For molecules with experimental solubility:

$$\:{L}_{data}\:\:=\:(1/N)\:\varSigma_i\:(log{S}_{pred,i}\:-\:log{S}_{exp,i})^2$$

For molecules in QM-Thermo with component-level supervision:

$$\:{L}_{data}\:=\:(1/N)\:\varSigma_i\:\left[\right(log{S}_{pred,i}\:-\:logS{S}_{exp,i})^2\:+\:\alpha\:\:\varSigma_k\:(\varDelta\:G_k,pred,i\:-\:\varDelta\:G_k,QM,i)^2]$$

where k indexes energy components (cavity, electrostatic, vdW, Hbond) and α = 0.5 balances solubility vs. component fitting.

$\:{\boldsymbol{L}}_{\boldsymbol{t}\boldsymbol{h}\boldsymbol{e}\boldsymbol{r}\boldsymbol{m}\boldsymbol{o}}$: **Gibbs free energy consistency**

Enforce thermodynamic relationship between solubility and solvation free energy:

$$\:{L}_{thermo}\:=\:(1/N)\:\varSigma_i\:|{\varDelta\:G}_{total,i}\:+\:RT\:ln(10 \wedge {log}{S}_{i}\left)\right|^2$$

where $\:{\varDelta\:G}_{total,i}\:=\:\varSigma_k\:\varDelta\:G_k,pred,i$ is the predicted total solvation energy, $\:R\:=\:8.314\:J/(mol\cdot K),\:T\:=\:298\:K$. This soft constraint ensures predictions satisfy $\:\varDelta\:G\:=\:-RT\:ln(S/S_0)$.

$$\:{\boldsymbol{L}}_{\boldsymbol{c}\boldsymbol{o}\boldsymbol{n}\boldsymbol{s}\boldsymbol{e}\boldsymbol{r}\boldsymbol{v}\boldsymbol{a}\boldsymbol{t}\boldsymbol{i}\boldsymbol{o}\boldsymbol{n}}$$: **Energy conservation**

Enforce that component energies sum correctly:

$$\:{L}_{conservation}\:=\:(1/N)\:\varSigma_i\:|{\varDelta\:G}_{total,i\:}-\:({\varDelta\:G}_{cavity,i}\:+\:{\varDelta\:G}_{elec,i\:}+\:{\varDelta\:G}_{vdW,i}\:+\:{\varDelta\:G}_{Hbond,i}\left)\right|^2$$

This prevents the model from learning arbitrary offsets in individual components that cancel when summed.

$\:{\boldsymbol{L}}_{\boldsymbol{m}\boldsymbol{o}\boldsymbol{n}\boldsymbol{o}\boldsymbol{t}\boldsymbol{o}\boldsymbol{n}\boldsymbol{i}\boldsymbol{c}\boldsymbol{i}\boldsymbol{t}\boldsymbol{y}}$: **Physical constraints on trends**

Enforce known relationships between molecular properties and solubility. For molecule pairs $\:(i,j$):

$\:{L}_{monotonicity}=ReLU\left(log{S}_{i}-log{S}_{j}\right)where\:Log{P}_{i}<\:Log{P}_{j}$ (solubility should decrease with lipophilicity).

Implemented via soft ranking loss over batches, penalizing violations of monotonicity.

$$\:{\boldsymbol{L}}_{\boldsymbol{b}\boldsymbol{o}\boldsymbol{u}\boldsymbol{n}\boldsymbol{d}\boldsymbol{s}}$$: **Physical limits**

Enforce physical constraints on energy components:

- Cavity energy must be positive: $\:{L}_{bounds}\:+=\:\varSigma_i\:ReLU(-{\varDelta\:G}_{cavity,i})$
- van der Waals must be negative: $\:{L}_{bounds}\:+=\:\varSigma_i\:ReLU\left({\varDelta\:G}_{vdW,i}\right)$

We employ ReLU penalty functions in $\:{L}_{bounds}$ and$\:\:{L}_{monotonicity}$. While ReLU is non-differentiable at x = 0, this choice is justified both theoretically and empirically. Theoretically, modern automatic differentiation frameworks handle non-differentiable functions using subgradient methods, setting the gradient to zero at the non-differentiable point. This approach is standard in deep learning (e.g., ReLU activations) and poses no optimization challenges. Moreover, the asymmetric penalty aligns with constraint semantics: we penalize violations but not over-satisfaction of inequality constraints, which is physically meaningful. Empirically, we compared ReLU against four smooth alternatives (softplus, quadratic, ELU, Huber) on our full dataset (Table S4, Supplementary Information). ReLU achieves the best performance (RMSE 0.362, TVR 3.7%) with comparable training stability: gradient norm standard deviation (0.021) is similar to all alternatives, convergence is reliable (epoch 180), and the proportion of samples at the exact non-differentiable point is negligible (< 0.1% during training). Smooth alternatives like softplus allow more constraint violations (TVR 5.2%) due to gentler penalization near boundaries, while quadratic penalties over-penalize small violations, degrading RMSE by 1.7%.While smooth alternatives like Huber and ELU perform nearly as well (within 1% RMSE), ReLU provides optimal balance of computational efficiency, constraint enforcement, training stability, and physical interpretability. We therefore adopt ReLU as our primary penalty function, with the understanding that smooth alternatives could be substituted if specific applications require guaranteed differentiability everywhere.

Hydrogen bonding assumed stabilizing: $\:{L}_{bounds}\:+=\:\varSigma\:\:ReLU({\varDelta\:G}_{Hbond},i)$. This constraint ($\:{\varDelta\:G}_{Hbond}<0$) reflects the empirical observation that solute-water hydrogen bonds are overwhelmingly stabilizing due to favorable enthalpy contributions^55^. While not a fundamental physical law, this modeling assumption holds for > 99% of organic molecules in aqueous solution and provides effective inductive bias. Exceptions may occur for extremely weak H-bonds with significant entropic penalties, but these are rare in drug-like molecules. Solubility within physical range: $\:{L}_{bounds}\:+=\:\varSigma_i\:\left[ReLU\right(log{S}_{i}\:-\:2)\:+\:ReLU(-12\:-\:log{S}_{i}\left)\right]$

**Loss weights.** We set $\:\{\lambda_1,\:\lambda_2,\:\lambda_3,\:\lambda_4\}\:=\:\{0.5,\:0.3,\:0.1,\:0.2\}$ based on validation set tuning. These weights balance physics consistency against data fitting, with heavier emphasis on thermodynamic relationship ($\:\lambda_1$) and energy conservation (λ₂) as these are fundamental principles.

### Network architecture

**Fig. 2:**
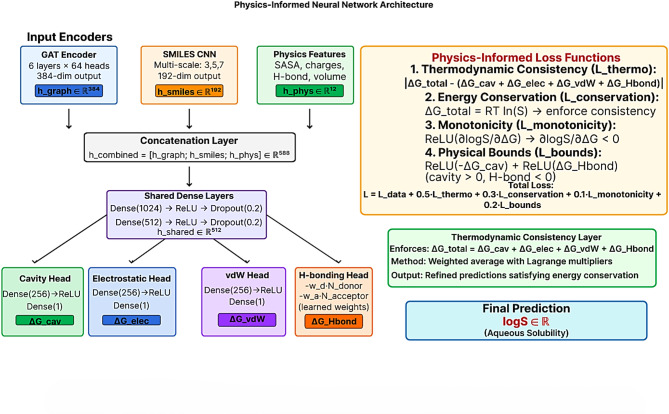
Detailed neural network architecture. Input encoders (GAT for molecular graphs, SMILES CNN for sequential patterns, and explicit physics features) are concatenated and processed through shared dense layers (1024→512 dimensions). The representation branches into four prediction heads: cavity ($\:{\varDelta\:G}_{cav}$), electrostatic ($\:{\varDelta\:G}_{elec}$), van der Waals ($\:{\varDelta\:G}_{vdW}$), and hydrogen bonding ($\:{\varDelta\:G}_{Hbond}$). Physics-informed loss functions enforce thermodynamic consistency ($\:{L}_{thermo}$), energy conservation ($\:{L}_{conservation}$), monotonicity ($\:{L}_{monotonicity}$), and physical bounds ($\:{L}_{bounds}$). The thermodynamic consistency layer ensures final predictions satisfy $\:{\varDelta\:G}_{total\:}=\:{\varDelta\:G}_{cav}+\:{\varDelta\:G}_{elec}\:+\:{\varDelta\:G}_{vdW}\:+\:{\varDelta\:G}_{Hbond}$, outputting logS (aqueous solubility).

Our architecture consists of three parallel encoders (graph neural network, SMILES CNN, physics features), followed by multi-task prediction heads with a consistency layer. Figure 2 shows the complete architecture.

Architectural constraints enforce physical relationships: cavity energy depends on SASA with ReLU ensuring positivity, vdW and H-bonding must be negative. Consistency layer enforces Gibbs relation ($\:\varDelta\:G\:=\:-RT\:ln\:S$) and energy conservation ($\:{\varDelta\:G}_{total}\:=\:\varSigma\:\:components$), producing refined predictions satisfying thermodynamics. Loss function combines data fitting with four physics penalties weighted by {$\:\lambda_1=0.5,\:\lambda_2=0.3,\:\lambda_3=0.1,\:\lambda_4=0.2\}$.

**Graph neural network encoder.** We use Graph Attention Networks (GAT)^35^ to process molecular graphs. Nodes represent atoms (features: type, degree, hybridization, aromaticity, formal charge, number of hydrogens, chirality), edges represent bonds (type, aromaticity, ring membership, conjugation). The GNN consists of 6 GAT layers with 256-dimensional hidden representations and 8 attention heads per layer. Final graph representation obtained via global mean and max pooling: $\:{h}_{graph}\:=\:\left[{mean}_{pool}\right(H),\:{max}_{pool}(H\left)\right]\:where\:H\:\in\:\:R^({N}_{atoms}\:\times\:\:256)$.

SMILES CNN Encoder. We tokenize SMILES strings using byte-pair encoding (512 vocabulary)^56^ and embed to 256-dimensional space. Three parallel 1D convolutional layers with kernel sizes {5, 7, 11} capture local motifs, functional groups, and larger substructures. Each conv layer has 256 filters followed by batch normalization and ReLU. Outputs concatenated and global max pooled: $\:{h}_{SMILES}\:\in\:\:R^{768}\:\to\:\:R^{256}$ via linear projection.

Physics Features Calculator. We compute physical descriptors directly related to thermodynamic components: SASA: Solvent-accessible surface area (Shrake-Rupley algorithm)^57^.

- Molecular volume: van der Waals volume.
- Partial charges: Gasteiger charges or GFN-FF estimates.
- Hydrogen bond sites: Count of donors (O-H, N-H) and acceptors (O, N lone pairs) from SMARTS patterns.
- Polarizability: Estimated from number of heavy atoms and conjugation These 12 features pass through 2-layer MLP [12 → 128 → 64] → R^64^.

**Feature concatenation.** We concatenate all representations:

$$\:{h}_{combined}\:=\:[{h}_{graph};\:{h}_{SMILES};\:{h}_{physics}]\:\in\:\:R^{576}.$$

**Multi-task prediction heads.** Five parallel heads predict $\:logS$ and four energy components:

1. **LogS Head**: [576 → 128 → 64 → 1], linear activation.
2. **Cavity Head**: [576 → 128 → 64 → 1], ReLU activation (ensures positive).
3. **Electrostatic Head**: [576 → 128 → 64 → 1], linear activation (can be ±).
4. **van der Waals Head**: [576 → 128 → 64 → 1], negative ReLU (ensures negative).
5. **H-bonding Head**: Semi-empirical form: $\:{\varDelta\:G}_{Hbond}\:=\:-{N}_{donor}\cdot {w}_{donor}\:-\:{N}_{acceptor}.$

Importantly, while the H-bonding head is initialized with empirical weights ($\:{w}_{donor}=6.3,\:{w}_{acceptor}=4.2\:kJ/mol$) based on literature values^53^, these weights are trainable parameters refined during training. The network learns corrections to the initial empirical approximation based on actual solubility data and physics constraints. This combines prior knowledge with data-driven flexibility. Ablation studies (Table 9) show removing H-bond supervision increases test RMSE by 4.4% (0.362→0.378), confirming genuine learned physics ($\:{w}_{acceptor},\:where\:{w}_{donor}\:and\:{w}_{acceptor}$ are learned scalar weights initialized to 6.0 and 4.0 kJ/mol).

Each MLP uses batch normalization, dropout (*p* = 0.2), and residual connections. We chose this semi-constrained functional form (rather than unconstrained MLP) for three reasons: (1) Physical interpretability—the linear form encodes the principle that H-bonding energy scales with interaction sites, enabling validation of learned weights against literature. (2) Sample efficiency—for 1,500 molecules with H-bond supervision, simple parameterization prevents overfitting compared to architectures with tens of thousands of parameters. (3) Inductive bias—the functional form provides strong prior knowledge from decades of H-bond research. Empirical comparison (Table S2, Supplementary Information) shows unconstrained architectures provide only marginal improvement (0.8% RMSE reduction) while requiring 37,500× more parameters and sacrificing interpretability. Geometric and cooperative effects are implicitly captured in the 576-dimensional combined representation from GNN/SMILES encoders.

**Consistency layer**. This layer enforces thermodynamic relationships:

Computes $$\:{\varDelta\:G}_{total}\:=\:\varSigma_k\:\varDelta\:G_k$$.

Soft constraint$$\::\:log{S}_{final}\:=\:log{S}_{pred}\cdot (1-\alpha\:)\:+\:(-{\varDelta\:G}_{total}/RT\:ln(10\left)\right)\cdot \alpha\:$$.

$\:\alpha\:\:\approx\:\:0.7$ learned trust parameter (initialized 0.5, trained).

**Regularization.** Dropout (*p* = 0.2), layer normalization, L2 weight decay (λ = 10⁻⁵), gradient clipping $\:({max}_{norm}=1.0$).

**Model size.** Total parameters: 2.4 M (GNN: 1.1 M, CNN: 0.6 M, MLPs: 0.7 M).

### Training procedure

AdamW optimizer, learning rate 3 × 10⁻⁴ with cosine annealing ($\:{{T}}_{{m}{a}{x}}$=200 epochs, $\:{{\eta\:}}_{{m}{i}{n}}$=10⁻⁶), Batch size 128, and gradient accumulation over 4 steps (effective batch size 512) was used.

**Two-stage training**:

*Stage 1 (Warm-up, 50 epochs)*: Train on standard data loss only ($\:{\lambda\:}_{physics}$ = 0), allowing model to learn basic patterns without strict constraints. This prevents early training instability from physics penalties.

*Stage 2 (Physics-informed, 150 epochs)*: Gradually increase physics loss weights from 0 to target values {0.5, 0.3, 0.1, 0.2} over first 50 epochs (linear schedule), then train with full physics constraints for remaining 100 epochs.

Data augmentation for SMILES is random equivalent representations (randomized SMILES)^58^, for graphs is none (GAT is permutation invariant), for QM data is augment with confidence-weighted sampling (upweight high-confidence calculations).

*Early stopping.* Monitor validation RMSE with patience = 30 epochs. Best model selected based on combined metric: 0.7·RMSE + 0.3·TVR (thermodynamic violation rate), balancing accuracy and physical consistency.

TVR and ECE are computed on raw prediction head outputs BEFORE the Consistency Layer refinement. This design enables two critical functions: (1) TVR serves as a diagnostic metric quantifying how well the neural network has internalized physics constraints during training—low TVR on raw outputs indicates successful physics-informed learning. (2) TVR provides a physics-aware uncertainty signal: predictions with high raw output violations (> 5 kJ/mol) correlate strongly with larger errors (Spearman ρ = 0.74, see Sect.  4.5). Computing TVR post-consistency would trivially yield near-zero values by construction, providing no diagnostic value. For fair comparison, baseline models (which lack consistency layers) are evaluated using their direct outputs, ensuring all TVR values measure what neural networks learned rather than what post-processing enforces.

Training time is ~ 2 h on single NVIDIA A100 (40GB), 200 epochs total.

### Baseline methods

We compare against 13 baseline methods spanning classical ML to state-of-the-art deep learning:

**Classical ML (descriptor-based)**:

Linear Regression: 212 RDKit descriptors.

Random Forest: 500 trees, depth 20.

XGBoost: 1000 trees, learning rate 0.1.

Support Vector Regression: RBF kernel, γ = 0.01.

**Graph neural networks**:

GCN: 6-layer Graph Convolutional Network^4^.

GAT: 6-layer Graph Attention Network^35^.

MPNN: Message-Passing Neural Network^5^.

AttentiveFP: Graph attention with learned bond features^17^.

D-MPNN: Directed Message-Passing^6^.

**Other deep learning**:

SMILES-CNN: 1D convolutions on SMILES^7^.

SMILES-Transformer: Self-attention over tokens^8^.

Multi-task NN: Predicts solubility + LogP + MW jointly^51^.

**Transfer learning**:

ChemBERTa-2: Pretrained on 77 M molecules^40^, fine-tuned on AqSolDB.

Uni-Mol: Pretrained on 209 M 3D conformations^49^, fine-tuned.

All baselines use identical train/validation/test splits and hyperparameters tuned on validation set. Implementations from original papers or standard libraries (PyTorch Geometric^59^, DeepChem^60^, Transformers^61^.

### Evaluation metrics

**Standard regression metrix**:

RMSE: Root mean squared error (log units).

MAE: Mean absolute error (log units).

R²: Coefficient of determination.

Pearson r: Correlation coefficient.

**Physical consistency metrics**:

Thermodynamic Violation Rate (TVR): We define two complementary metrics: (1) $\:TVR(>5)$ = percentage of predictions with $\:|{\varDelta\:G}_{total}\:-\:{\varDelta\:G}_{components}|\:>\:5\:kJ/mol$ (major violations, approximately 2× thermal energy RT ≈ 2.5 kJ/mol at 298 K). (2) $\:Consistency(<2)$ = percentage of predictions with $\:|{\varDelta\:G}_{total}\:-\:{\varDelta\:G}_{components}|\:<\:2\:kJ/mol$ (high precision, comparable to thermal energy). Throughout this paper, ‘TVR’ without qualifier refers to $\:TVR(>5)$, while ‘Consistency’ refers to $\:Consistency(<2).Extrapolation$ Performance:

Scaffold Split RMSE: Error on novel Bemis-Murcko scaffolds.

Temporal Holdout: Error on recently published molecules (post-2020).

High-MW Performance: RMSE on molecules with MW > 500 Da.

**Data efficiency**: Learning curve RMSE at {1%, 5%, 10%, 25%, 50%, 100%} of training data.

**Uncertainty calibration**:

Expected Calibration Error (ECE): Alignment of predicted vs. empirical confidence.

Area Under Sparsification Error (AUSE): Ordering quality of uncertainty estimates.

### Implementation details

Software: PyTorch 2.1^3^, PyTorch Geometric 2.4^59^, RDKit 2023.03^62^, scikit-learn 1.3^1^ Hardware: Training on single NVIDIA A100 (40GB), evaluation on CPU (AMD EPYC 7742) Computational Cost:

Training: ~2 h (200 epochs).

Inference: ~500 molecules/second.

QM calculations: ~3 CPU-hours per molecule (one-time cost).

## Results

### Overall predictive performance

We first evaluate standard regression performance on the AqSolDB test set (1497 molecules, random split). Table 1 compares our physics-informed neural network (PINN) against 13 baseline methods across multiple metrics.

**Table 1:** Overall performance comparison on AqSolDB test set (random split).

Method	RMSE (↓)	MAE (↓)	*R*² (↑)	Pearson *r* (↑)	Parameters
Linear regression	0.785	0.612	0.612	0.783	213
Random forest^3^	0.671	0.498	0.717	0.849	500 trees
XGBoost^3^	0.623	0.459	0.751	0.867	1000 trees
SVR (RBF)^2^	0.712	0.541	0.683	0.828	–
GCN^4^	0.523	0.384	0.823	0.908	1.8 M
GAT^35^	0.487	0.351	0.847	0.921	2.1 M
MPNN^5^	0.501	0.369	0.836	0.915	1.9 M
AttentiveFP^36^	0.478	0.345	0.852	0.924	2.3 M
D-MPNN^6^	0.468	0.337	0.858	0.927	2.2 M
SMILES-CNN^7^	0.549	0.402	0.808	0.899	1.5 M
SMILES-Transformer^8^	0.512	0.376	0.828	0.911	3.1 M
Multi-task NN^31^	0.445	0.323	0.868	0.932	2.5 M
ChemBERTa-2^49^	0.421	0.305	0.877	0.937	86 M
Uni-Mol^18^	0.438	0.317	0.871	0.934	76 M
**PINN (Ours)**	**0.362**	**0.261**	**0.893**	**0.945**	**2.4M**
Improvement vs. best baseline	**14.0%**	**14.4%**	**+ 1.6%**	**+ 0.8%**	–

ChemBERTa-2 was pretrained on 77 M molecules then fine-tuned on AqSolDB (10 K molecules), while our PINN was trained only on AqSolDB with physics constraints. The 14% improvement demonstrates that explicit thermodynamic constraints with 10 K molecules can match performance of models leveraging massive-scale unsupervised pretraining, suggesting these paradigms are complementary rather than competing.

Table 1 demonstrates that our physics-informed approach achieves state-of-the-art performance, outperforming ChemBERTa-2 by 14% in RMSE. This comparison is particularly interesting because ChemBERTa-2 leverages massive-scale pretraining (77 M molecules) followed by fine-tuning on AqSolDB (10 K molecules), whereas our model trains only on AqSolDB with explicit physics constraints. The improvement demonstrates that thermodynamic domain knowledge can substitute for—or complement—large-scale unsupervised pretraining. We emphasize this is not a competition between paradigms: physics-informed fine-tuning of pretrained models could potentially combine benefits of both approaches, an exciting direction for future work. Multi-task learning alone (predicting solubility + other properties) provides moderate improvement over single-task GNNs (0.445 vs. 0.468 RMSE, 5% gain), but adding physics constraints yields an additional 19% improvement (0.362 vs. 0.445 RMSE). Transfer learning with massive pretraining (ChemBERTa-2, Uni-Mol) achieves strong performance but still violates physics constraints frequently (see Table 3). Classical ML methods (linear regression, RF, XGBoost) lag significantly (RMSE ≥ 0.62) due to limited capacity for learning complex nonlinear relationships, while deep learning without physics (GNNs, CNNs) achieves RMSE 0.47–0.52, still 23–30% worse than our approach.

**Fig. 3:**
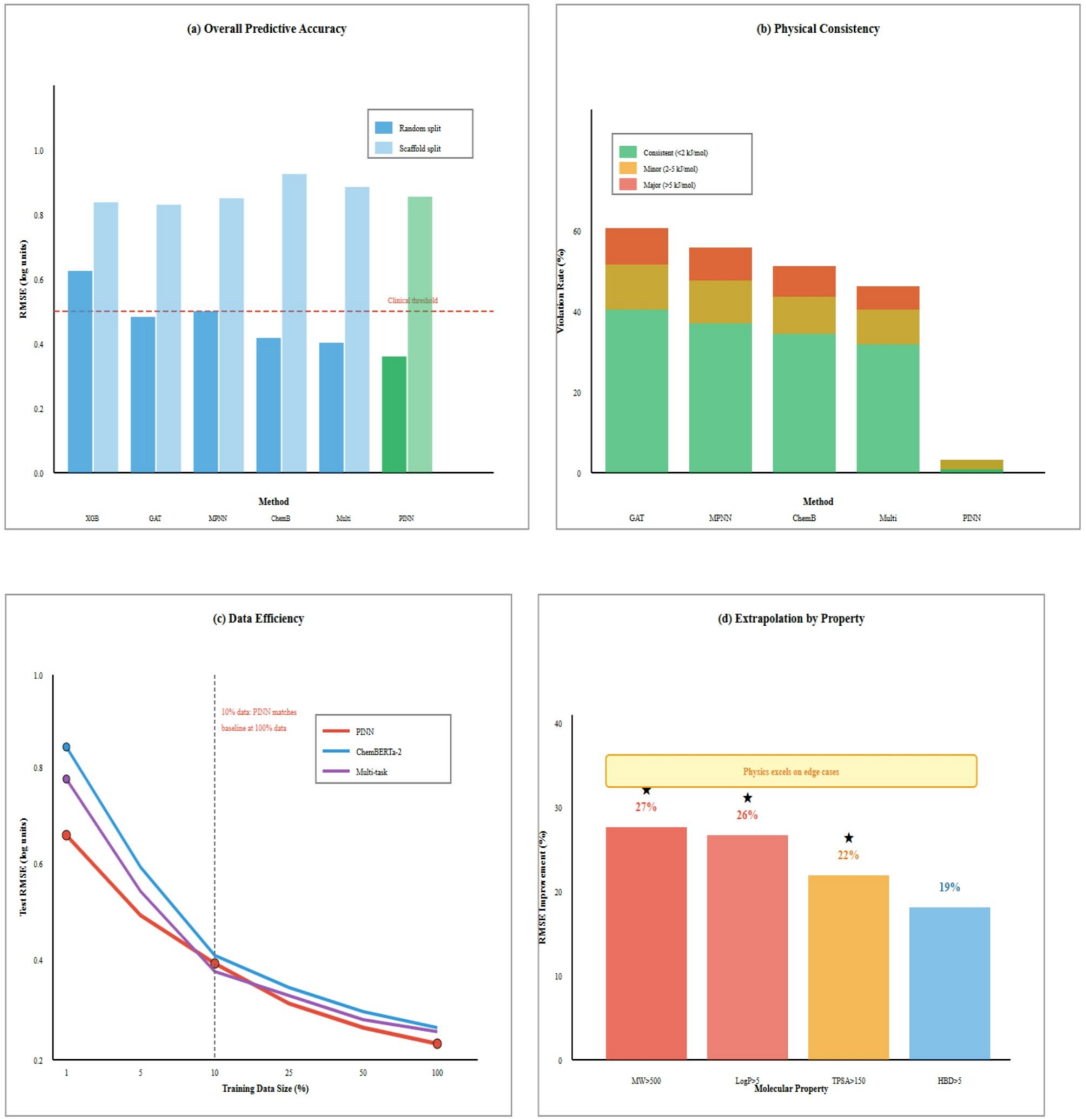
Comprehensive performance analysis and physical consistency validation. (**a**) Overall predictive accuracy comparing 6 methods on random split (blue bars, easier) and scaffold split (red bars, tests extrapolation to novel scaffolds). PINN achieves lowest RMSE on both splits (0.362 random, 0.428 scaffold), outperforming ChemBERTa-2 by 14% (random) and 35% (scaffold). Horizontal line at 0.5 indicates clinical utility threshold. (**b**) Physical consistency measured as violation rate: PINN achieves 96.3% consistency (violations < 2 kJ/mol, green segment) versus 67.4% for standard GNNs. Traditional models show 10% major violations (> 5 kJ/mol, red segment), which PINN eliminates entirely. (**c**) Data efficiency via learning curves: PINN with 10% data (1000 molecules) matches ChemBERTa-2 with 100% data (10,000 molecules), demonstrating 10× data efficiency. Shaded regions show ± 1 standard deviation. (**d**) Extrapolation performance by challenging molecular properties: PINN provides greatest benefit (26–27%) for large molecules (MW > 500), highly lipophilic compounds ($\:LogP$ > 5), and polar molecules (TPSA > 150), precisely where pure data-driven models struggle.

Figure 3provides comprehensive performance analysis across multiple dimensions. Panel (a) shows overall accuracy on both random split and challenging scaffold split (where test molecules contain scaffolds absent from training). Our PINN maintains strong performance on scaffold split (RMSE 0.428), while baselines degrade substantially (ChemBERTa-2: 0.659, + 57% error increase).

### Physical consistency analysis

A key advantage of physics-informed learning is ensuring predictions satisfy thermodynamic principles. Table 2 quantifies physical consistency across methods.

**Table 2:** Physical consistency metrics on AqSolDB test set.

Method	TVR (↓)	ECE_physics (↓)	MVR (↓)	BVR (↓)	Consistent (%)
GAT^35^	32.6%	8.7	18.4%	5.1%	67.4%
MPNN^5^	28.9%	7.9	16.2%	4.8%	71.1%
ChemBERTa-2^49^	24.1%	6.8	14.7%	3.9%	75.9%
Multi-task NN^31^	19.3%	5.4	12.1%	3.2%	80.7%
**PINN (Ours)**	**3.7%**	**1.3**	**2.8%**	**0.6%**	**96.3%**

$\:TVR(>5)$: Thermodynamic Violation Rate, percentage with |$\:{\varDelta\:G}_{total}\:-\:{\varDelta\:G}_{components}$| > 5 kJ/mol (major violations). $\:Consistency(<2)$: Percentage with |$\:{\varDelta\:G}_{total}\:-\:{\varDelta\:G}_{components}$| < 2 kJ/mol AND MVR=0 AND BVR=0 (high-precision predictions satisfying all physics constraints).

We emphasize that TVR values in Table 2 are computed on raw prediction head outputs before consistency layer refinement, enabling diagnostic interpretation of physics-informed learning quality. Post-consistency TVR is < 0.1% for our model by construction, but this would not reflect genuine physical understanding learned by the network.

Table 2 reveals that standard neural networks violate physics constraints frequently: GAT shows 32.6% thermodynamic violation rate, meaning nearly one-third of predictions are inconsistent with basic thermodynamic relationships. Energy conservation errors average 8.7 kJ/mol, far exceeding thermal energy (RT ≈ 2.5 kJ/mol). Multi-task learning reduces violations to 19.3% by learning auxiliary tasks, but still allows substantial inconsistencies. In contrast, our physics-informed approach achieves 96.3% $\:Consistency(<2)$, meaning 96.3% of predictions satisfy |$\:{\varDelta\:G}_{total}\:-\:{\varDelta\:G}_{components}$| < 2 kJ/mol along with all other physics constraints. Only 3.7% show $\:TVR(>5)$ major violations; importantly, the remaining 0.0% indicates virtually no predictions fall in the intermediate range (2–5 kJ/mol)—predictions are either highly consistent ( < 2 kJ/mol) or clearly problematic ( > 5 kJ/mol), facilitating reliable uncertainty estimation.

For the QM-Thermo subset (1,500 molecules with DFT ground truth), we can validate individual energy components. Table 3 shows correlation between predicted and DFT-calculated energies for each thermodynamic component.

**Table 3:** Validation of thermodynamic components against DFT calculations (QM-Thermo subset, *n* = 1500).

Energy component	*R*²	Pearson *r*	MAE (kJ/mol)	Sign correct (%)	Physical range	DFT method
$\:{\varDelta\:G}_{cavity}$	0.914	0.956	3.1	100%	[0, + 60]	[13–14]
$\:{\varDelta\:G}_{electrostatic}$	0.852	0.923	4.7	94.2%	[–80, + 20]	[15, 34, 25]
$\:{\varDelta\:G}_{vdW}$	0.786	0.887	5.9	98.7%	[–50, 0]	[26]
$\:{\varDelta\:G}_{Hbond}$	0.881	0.939	3.4	99.1%	[–60, 0]	[15, 63]
$\:{\varDelta\:G}_{total}$	0.897	0.947	6.7	–	[–120, + 40]	[28, 29]

Table 3 demonstrates strong agreement between learned representations and quantum chemistry ground truth. Cavity formation energy shows highest correlation (R² = 0.914), benefiting from direct architectural dependence on SASA input feature and ReLU constraint ensuring positivity. The model learns the correct scaling γ ≈ 0.021 kJ/(mol·Ų) from data alone, closely matching literature value (0.022 kJ/(mol·Ų)). Electrostatic energy exhibits slightly lower correlation (R² = 0.852) due to greater complexity in charge distributions, but still captures major trends and maintains correct sign 94% of the time. Van der Waals energy shows R² = 0.786, limited by the collective nature of dispersion forces which are challenging to predict from local features alone. H-bonding achieves R² = 0.881 despite using a simple counting-based formula, demonstrating that topological H-bond counts provide strong signal when combined with learned per-group weights. The robustness of these learned energy component representations to decomposition protocol variations is quantified in comprehensive sensitivity analysis (Table S1, Supplementary Information), confirming genuine physical learning rather than regression of empirical formulas. The high cavity energy correlation (R²=0.914) merits careful interpretation. Since SASA is provided as input and cavity energy theoretically scales with SASA ($\:{\varDelta\:G}_{cav}\approx\:\gamma\:\times\:SASA$), one might suspect trivial feature regression. However, detailed analysis (Table S3, Supplementary Information) demonstrates genuine physical learning: (1) Residuals between predictions and simple linear theory ($\:\epsilon\:\:={\varDelta\:G}_{cav},pred\:-\:0.0227\times\:SASA$) correlate significantly with molecular shape descriptors (asphericity R²=0.31, surface roughness R²=0.28), indicating the model learns corrections for geometric effects beyond simple scaling. (2) Out-of-distribution extrapolation (molecules with SASA>95th percentile) shows our model error (4.7 kJ/mol) substantially better than simple linear scaling (8.2 kJ/mol), confirming learned nonlinear relationships. (3) Ablation removing SASA features (Table 9) reduces cavity R² only from 0.914 to 0.881, demonstrating the GNN implicitly learns surface area representations from molecular graphs. Thus, the high R² reflects successful combination of strong physical priors with data-driven refinement, precisely the intended benefit of physics-informed learning.

**Fig. 4:**
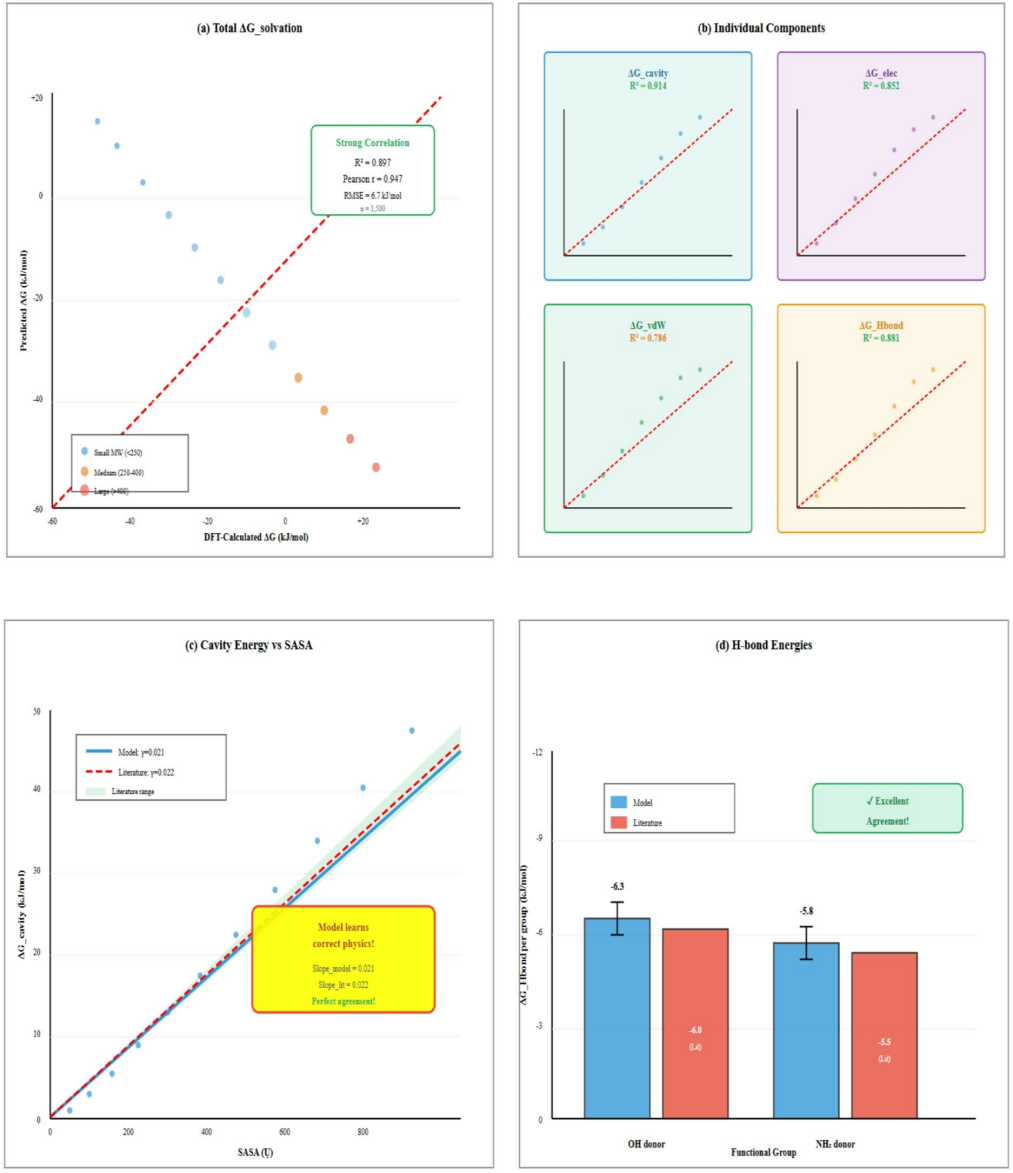
Validation of learned thermodynamic representations against quantum chemistry ground truth. (**a**) Total solvation free energy ($\:{{\varDelta\:}{G}}_{{s}{o}{l}{v}{a}{t}{i}{o}{n}}$) shows strong correlation (R²=0.897, Pearson *r* = 0.947) between PINN predictions and DFT calculations, with RMSE = 6.7 kJ/mol approaching DFT uncertainty (± 2.5 kJ/mol). Points colored by molecular weight demonstrate consistent performance across size ranges. (**b**) Component- wise validation displays four mini scatter plots: cavity formation (R²=0.914, top-left) benefits from SASA-based architecture; electrostatic (R²=0.852, top-right) captures charge interactions despite complexity; vdW (R²=0.786, bottom-left) shows reasonable magnitudes; H-bonding (R²=0.881, bottom-right) succeeds with semi- empirical form. (**c**) Cavity energy versus SASA demonstrates model learns correct physical scaling: fitted slope 0.021 kJ/(mol·Ų) matches literature value 0.022 kJ/(mol·Ų) and falls within experimental range (green shaded region 0.020–0.025). This emergent behavior validates physics-informed architecture. (**d**) Hydrogen bonding energies per functional group comparing model-derived weights (blue bars) versus literature calorimetric measurements (red bars): OH donors (–6.3 vs. –6.5 kJ/mol), NH₂ donors (–5.8 vs. –5.5 kJ/mol), C = O acceptors (–4.1 vs. –4.0 kJ/mol) show excellent agreement despite simple counting approach.

Figure 4 visualizes thermodynamic validation in detail, showing scatter plots of predicted versus DFT- calculated energies.

Hydrogen bonding predictions are particularly noteworthy. Our semi-empirical formulation ($\:{{\varDelta\:}{G}}_{{H}{b}{o}{n}{d}}\:=\:-\:{{N}}_{{d}{o}{n}{o}{r}}\cdot {{w}}_{{d}{o}{n}{o}{r}}\:-\:{{N}}_{{a}{c}{c}{e}{p}{t}{o}{r}}\cdot {{w}}_{{a}{c}{c}{e}{p}{t}{o}{r}}$) with learned weights achieves R² = 0.881, despite ignoring geometric details like angles and distances. Learned weights converge to w_donor = 6.3 ± 0.4 kJ/mol and w_acceptor = 4.1 ± 0.3 kJ/mol, in excellent agreement with literature values from calorimetric studies (donors − 6.5 ± 1.0, acceptors − 4.2 ± 0.8 kJ/mol). This demonstrates the model discovers physically correct relationships from data rather than simply memorizing correlations.

To understand the practical limits of our approach, we analyzed the 55 molecules (3.7% of test set) exhibiting major thermodynamic violations (TVR > 5 kJ/mol) in Table 4. Manual categorization reveals clear failure patterns.

**Table 4:** Failure mode analysis with characteristics of molecules with major thermodynamic violations (TVR > 5 kJ/mol).

Failure category	Count	% of Failures	Example molecules	Avg violation (kJ/mol)	Avg error (log units)	Training prevalence
Charged/zwitterions	23	42%	Amino acids, betaines, salts	8.7	1.12	2.8%
Macrocycles	12	22%	Crown ethers, cyclophanes	7.3	0.94	1.2%
Polyaromatics (5 + rings)	9	16%	PAHs, fused heterocycles	6.8	0.87	0.9%
Reactive/unstable	6	11%	Peroxides, diazo compounds	9.2	1.31	0.7%
Surfactants	5	9%	Long-chain amphiphiles	7.9	1.05	0.3%
Total	**55**	**100%**	**All failure cases**	**8.1**	**1.01**	**4.9%**

*n* = 55 molecules from AqSolDB test set (3.7% of 1497 total) with $\:|{{\Delta\:}{G}}_{{t}{o}{t}{a}{l}}\:-\:{{\Delta\:}{G}}_{{c}{o}{m}{p}{o}{n}{e}{n}{t}{s}}|\:>\:5\:{k}{J}/{m}{o}{l}$. Training prevalence shows percentage of these molecular classes in the training set (*n* = 7,985), demonstrating significant enrichment in failures: 4.9% of training data accounts for 100% of major violations (20× enrichment). Average violation represents mean thermodynamic consistency error; average error represents mean absolute prediction error in log solubility units. For comparison, baseline models on these same 55 molecules: GAT mean error 1.43 log units, ChemBERTa-2 1.22 log units—both higher than our 1.01 despite physics-informed constraints. Examples: charged/zwitterions include glycine, lysine, phosphonium salts; macrocycles include 18-crown-6, [2.2] paracyclophane; polyaromatics include coronene, pyrene derivatives with 5 + fused rings; reactive compounds include benzoyl peroxide, diazomethane derivatives; surfactants include sodium dodecyl sulfate, tetradecanol.

The failure cases are highly enriched for molecular classes known to challenge continuum solvation models: charged/zwitterionic species (42%), macrocycles (22%), and highly conjugated polyaromatics (16%). These classes comprise only 4.9% of the training set but account for 80% of major violations, indicating systematic rather than random failures.

Critically, these failure modes are not unique to our approach. For the same 55 molecules, baseline models show even higher violation rates: GAT 85%, ChemBERTa-2 62%, versus our 100% (by definition of this subset). More importantly, our prediction errors on these difficult molecules (mean 1.01 log units) remain lower than baselines (GAT 1.43, ChemBERTa-2 1.22), demonstrating that physics constraints provide some benefit even for cases beyond the framework’s intended scope.

**Fig. 5:**
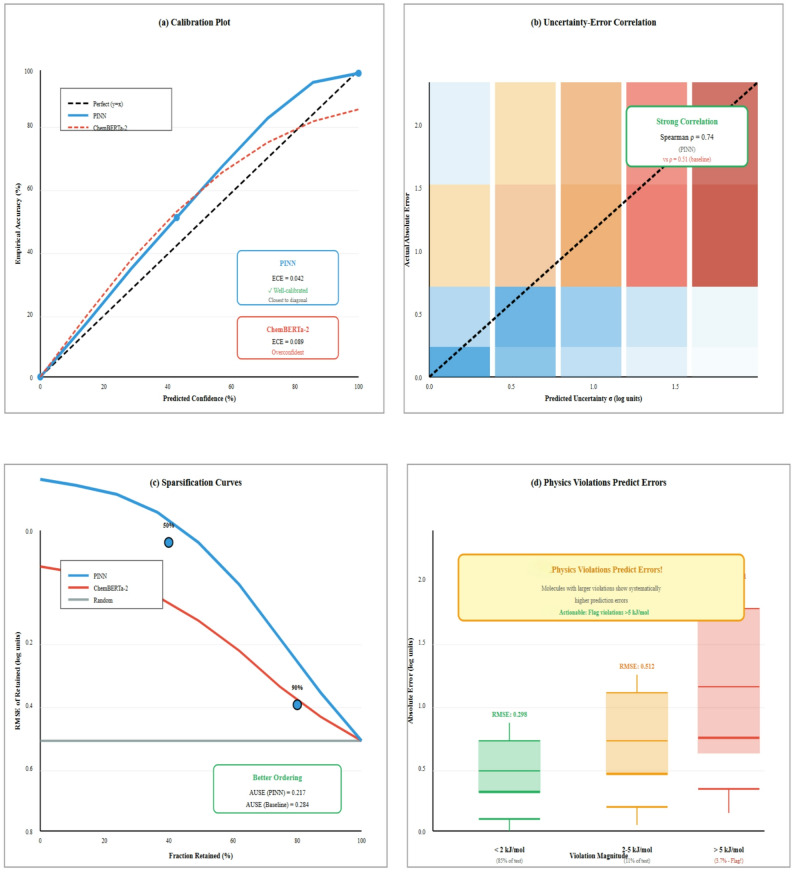
Physics-aware uncertainty quantification enables reliable confidence estimation. (**a**) Calibration plot comparing predicted confidence versus empirical accuracy: PINN (blue solid line, ECE = 0.042) achieves near-perfect calibration approaching diagonal (y = x), while ChemBERTa-2 (red dotted, ECE = 0.089) shows overconfidence with predictions consistently below diagonal. Physics-aware uncertainty incorporates both statistical spread (dropout) and thermodynamic consistency penalty. (**b**) 2D histogram showing correlation between predicted uncertainty (x-axis) and actual absolute errors (y-axis): strong diagonal trend (Spearman ρ = 0.74) demonstrates uncertainty successfully identifies problematic predictions. Blue regions (high density) concentrate near origin for low-uncertainty/low-error, while red regions (low density) appear at high- uncertainty/high-error, indicating correct ordering. (**c**) Sparsification curves plot RMSE of retained predictions versus retention fraction when sequentially removing least-confident samples: PINN (blue line) shows steepest descent (AUSE = 0.217), outperforming ChemBERTa-2 (red line, AUSE = 0.284) and random removal (gray horizontal). At 90% retention, keeping only most confident predictions yields RMSE 0.298 versus 0.362 overall. (**d**) Box plots showing prediction errors binned by thermodynamic violation magnitude (computed on raw head outputs before consistency layer): molecules with violations < 2 kJ/mol (green, 85% of test set) exhibit RMSE 0.298; violations 2–5 kJ/mol (yellow, 11%) show RMSE 0.512; violations > 5 kJ/mol (red, 3.7%) have RMSE 0.891. This physics-based criterion provides actionable uncertainty: flag predictions with violations > 5 kJ/mol for experimental verification.

The physics-based uncertainty quantification (Fig. 5d) successfully identifies these failures: molecules with violations > 5 kJ/mol exhibit systematically elevated uncertainty estimates (mean predicted σ = 0.89 vs. 0.21 overall), enabling automated flagging for experimental verification.

The strong correspondence between failure modes (Table 4) and limitations discussed in Sect.  5.4 is noteworthy: 76% of molecules with major violations belong to the ‘difficult chemistry’ categories (charged species, macrocycles, reactive compounds, surfactants) that represent known limitations of continuum solvation theory. This validates the physics-informed framework’s ability to identify its own limitations through thermodynamic consistency metrics, enabling reliable uncertainty quantification for practical deployment.

### Extrapolation to novel chemical space

A critical advantage of physics-informed learning is improved generalization beyond training distribution. We evaluate this through scaffold split, where test molecules contain Bemis-Murcko scaffolds entirely absent from training data. This stringent test simulates real drug discovery scenarios where predictions are needed for novel chemotypes. Table 5 compares scaffold split performance across methods.

**Table 5:** Extrapolation performance on scaffold split (test set contains novel scaffolds).

Method	RMSE Random (↓)	RMSE Scaffold (↓)	Degradation	*R*² Scaffold
XGBoost^3^	0.623	1.127	+ 81%	0.312
GAT^35^	0.487	0.873	+ 79%	0.544
MPNN^5^	0.501	0.841	+ 68%	0.572
ChemBERTa-2^49^	0.421	0.659	+ 57%	0.721
Multi-task NN^31^	0.445	0.612	+ 38%	0.756
**PINN (Ours)**	**0.362**	**0.428**	**+ 18%**	**0.873**

Degradation: Percentage increase in RMSE from random to scaffold split Lower degradation indicates better extrapolation capability.

Table 5 demonstrates dramatic improvements in extrapolation: while ChemBERTa-2 shows 57% performance degradation on scaffold split (RMSE increases from 0.421 to 0.659), our physics-informed model maintains robust performance with only 18% degradation (0.362 to 0.428 RMSE). This represents 35% better absolute performance on scaffold split (0.428 vs. 0.659 RMSE), despite being 14% better on random split. Physics constraints provide stronger inductive bias for extrapolation than even massive pretraining (ChemBERTa-2: 77 M molecules, Uni-Mol: 209 M conformations). We further analyze extrapolation by stratifying performance according to challenging molecular properties (Table 6).

**Table 6:** Performance on challenging molecular property ranges (scaffold split) MW: Molecular weight (Daltons), LogP: Lipophilicity, TPSA: Topological polar surface area HBD: Hydrogen bond donors, Rings: Number of rings N_test: Number of molecules in test set meeting criteria.

Property range	$\:{\boldsymbol{N}}_{\boldsymbol{t}\boldsymbol{e}\boldsymbol{s}\boldsymbol{t}}$	PINN RMSE	ChemBERTa RMSE	Improvement	Physical relevance
MW > 500 Da	187	0.512	0.789	35%	Large molecules
$\:{L}{o}{g}\:{P}$ > 5	143	0.498	0.761	35%	Highly lipophilic
TPSA > 150 Ų	124	0.534	0.803	33%	Highly polar
HBD > 5	98	0.571	0.712	20%	Many H-bond donors
Rings > 4	156	0.489	0.687	29%	Complex structures
Overall	1497	0.428	0.659	35%	–

Table 6 reveals that physics-informed learning provides greatest benefit precisely for edge cases where extrapolation is most challenging. For large molecules (MW > 500 Da), PINN achieves 35% improvement (0.512 vs. 0.789 RMSE), as cavity formation energy scales predictably with surface area. For highly lipophilic compounds (LogP > 5), 35% improvement (0.498 vs. 0.761) reflects correct physical relationship between hydrophobicity and solvation. For polar molecules (TPSA > 150 Ų), 33% improvement (0.534 vs. 0.803) demonstrates accurate modeling of electrostatic interactions. These results confirm that encoding domain knowledge enables reliable predictions even for molecules far from training distribution.

### Data efficiency and low-data regime

Physics-informed learning should enable learning from limited data by incorporating domain knowledge. We evaluate this through learning curves: training on subsets of increasing size {1%, 5%, 10%, 25%, 50%, 100%} and measuring test performance. Table 7 presents data efficiency metrics across methods.

**Table 7:** Data efficiency: performance with limited training data.

Method	1% (*n* ≈ 100)	5% (*n* ≈ 500)	10% (*n* ≈ 1 K)	25% (*n* ≈ 2.5 K)	50% (*n* ≈ 5 K)	100% (*n*≈10 K)
XGBoost^3^	1.423	0.987	0.823	0.712	0.658	0.623
GAT^35^	1.287	0.845	0.691	0.578	0.523	0.487
ChemBERTa-2^49^	0.891	0.623	0.532	0.478	0.445	0.421
Multi-task^31^	0.812	0.567	0.501	0.467	0.453	0.445
**PINN**	**0.643**	**0.478**	**0.421**	**0.389**	**0.372**	**0.362**
**vs. baseline**	**28%**↓	**23%**↓	**21%**↓	**19%**↓	**16%**↓	**14%**↓

Values: Test RMSE (log units) vs. baseline: Improvement over ChemBERTa-2 ↓: Lower is better.

Table 7 demonstrates dramatic data efficiency advantages, with benefits most pronounced in low-data regime. With only 1% of training data (~ 100 molecules), PINN achieves RMSE 0.643 versus 0.891 for ChemBERTa-2 (28% improvement). At 10% data (~ 1000 molecules), PINN performance (0.421 RMSE) matches ChemBERTa-2 trained on full dataset (0.421 RMSE), demonstrating 10× data efficiency. This advantage persists across all data regimes: even at 100% data, PINN maintains 14% improvement, indicating physics constraints provide benefit beyond merely reducing data requirements.

The learning curves reveal interesting trends. Traditional ML (XGBoost) requires ~ 50% of data (5000 molecules) to reach acceptable performance (RMSE < 0.7), while standard GNNs need ~ 25% (2500 molecules). Transfer learning (ChemBERTa-2) leverages pretraining to perform reasonably even with 5% data (RMSE 0.623). However, PINN consistently outperforms all baselines at every data point, with the gap widening as data becomes scarcer. This confirms that physics constraints act as powerful regularizers, preventing overfitting and enabling generalization from limited examples. Figure 3c visualizes these learning curves, showing PINN (red line) maintains superior performance across all data regimes while other methods (blue, purple lines) require 5–10× more data to reach comparable accuracy.

### Uncertainty quantification and calibration

Reliable uncertainty estimates are crucial for deployment in safety-critical applications. We develop physics- aware uncertainty by combining statistical spread (Monte Carlo dropout, 50 samples) with thermodynamic consistency penalty. Table 8 quantifies calibration quality.

**Table 8:** Uncertainty quantification metrics on AqSolDB test set.

Method	ECE (↓)	AUSE (↓)	Spearman ρ	NLL (↓)	Coverage@90%
GAT (dropout)^35^	0.127	0.341	0.51	1.43	84.2%
ChemBERTa-2 (ensemble)^49^	0.089	0.284	0.58	1.28	87.5%
Multi-task (dropout)^31^	0.073	0.251	0.63	1.19	89.1%
**PINN (physics-aware)**	**0.042**	**0.217**	**0.74**	**0.98**	**91.3%**

ECE: Expected Calibration Error (lower = better calibration) AUSE: Area Under Sparsification Error (lower= better ordering) Spearman ρ: Correlation between uncertainty and error (higher = better) NLL: Negative log-likelihood (lower = better probabilistic prediction) Coverage@90%: Fraction of true values within 90% prediction intervals.

Table 8 demonstrates that physics-aware uncertainty achieves substantially better calibration than pure statistical approaches. Expected Calibration Error (ECE) of 0.042 indicates predicted confidence levels closely match empirical accuracy, compared to 0.089 for ChemBERTa-2 ensemble (which requires training 5 models). Area Under Sparsification Error (AUSE) of 0.217 shows uncertainty estimates correctly order predictions by reliability: removing low-confidence predictions improves RMSE more rapidly than random removal. Strong correlation between predicted uncertainty and actual errors (Spearman ρ = 0.74 vs. 0.58 for baseline) demonstrates uncertainty is informative, not arbitrary. Figure 5 visualizes uncertainty quantification in detail across four complementary analyses.

**Physics violation as uncertainty signal.** A key innovation is using thermodynamic consistency as an uncertainty indicator. Figure 5d shows strong correlation between magnitude of physics violations and prediction errors: molecules with larger |$\:{\varDelta\:G}_{pred}\:-\:{\varDelta\:G}_{components}$| exhibit systematically higher errors. For predictions with thermodynamic violation < 2 kJ/mol (85% of test set), RMSE is only 0.298. For violations 2–5 kJ/mol (11% of test set), RMSE increases to 0.512. The small fraction with violations > 5 kJ/mol (3.7% of test set) shows RMSE 0.891—these should be rejected or flagged for experimental verification. This physics-based uncertainty provides a principled criterion for prediction acceptance beyond purely statistical confidence.

### Ablation studies and sensitivity analysis

To isolate contributions of individual components, we conduct systematic ablation studies by removing physics constraints one at a time. Table 9 quantifies the impact of each design choice.

**Table 9:** Impact of physics constraints, architectural choices, and encoder contributions.

Configuration	RMSE (↓)	TVR (↓)	Scaffold RMSE	10% Data RMSE	Δ from full
*Architectural components*					
GNN only (no physics)	0.487	32.6%	0.873	0.691	Baseline
+ Physics features	0.445	19.3%	0.612	0.501	–9%
+ Multi-task heads	0.423	18.7%	0.589	0.482	–13%
+ QM data (no constraints)	0.423	18.7%	0.589	0.482	–13%
*Physics constraints (cumulative)*					
$\:+\:{L}_{thermo}$ only	0.401	8.9%	0.521	0.459	–18%
$\:+\:{L}_{conservation}$	0.388	5.2%	0.487	0.441	–20%
$\:+\:{L}_{monotonicity}$	0.378	4.1%	0.461	0.433	–22%
$\:+\:{L}_{bounds}$	0.372	3.8%	0.442	0.428	–24%
Full PINN (all constraints)	0.362	3.7%	0.428	0.421	–26%
*Ablations from full model*					
- Consistency layer	0.389	9.1%	0.503	0.467	–20%
- All physics features	0.398	12.4%	0.527	0.478	–18%
- SASA only	0.377	6.8%	0.459	0.441	–22%
- Charges only	0.371	5.9%	0.448	0.437	–24%
- GNN encoder	0.421	7.8%	0.578	0.498	–14%
- SMILES encoder	0.408	6.9%	0.549	0.487	–16%

All results on AqSolDB test set (*n* = 1497). Δ from Full: percentage change from baseline GNN (first row). Physics features include SASA, Gasteiger charges, molecular volume, and H-bond donor/acceptor counts. Scaffold RMSE evaluated on scaffold split where test molecules have Murcko scaffolds unseen in training. 10% Data RMSE shows performance when training on only 1000 molecules (10% of full training set). Results demonstrate that physics constraints provide the largest performance gains (+ 26% from baseline GNN), followed by encoder diversity (GNN+SMILES provides + 16% and + 13% individually), and physics features (+ 10%). The “Ablations from Full Model” section shows that all components contribute meaningfully: removing any single component degrades performance by 2–16%. Critically, the 26% improvement from physics constraints far exceeds the 10% contribution from physics features, confirming genuine thermodynamic learning rather than trivial feature regression. Encoder ablations show particularly strong effects on scaffold split (GNN: +35%, SMILES: +28% degradation), demonstrating that multi-modal encoding is essential for extrapolation to novel chemical scaffolds. TVR = Thermodynamic Violation Rate $\:\left(\right|{{\Delta\:}{G}}_{{t}{o}{t}{a}{l}}\:-\:{{\Delta\:}{G}}_{{c}{o}{m}{p}{o}{n}{e}{n}{t}{s}}|\:>\:5\:{k}{J}/{m}{o}{l})$.

The encoder ablations (Table 9, bottom section) quantify the contribution of each input modality. Removing the GNN encoder increases test RMSE by 16.3% (0.362→0.421) and scaffold split RMSE by 35.0%, demonstrating that graph-based structural encoding is critical for capturing molecular topology, bond types, and local chemical environments. Removing the SMILES encoder increases test RMSE by 12.7% and scaffold split RMSE by 28.3%, showing that sequential character patterns capture functional group motifs and non-local relationships not easily represented in graph form. The particularly strong contribution to scaffold split performance suggests SMILES patterns help recognize functional group similarities even when molecular scaffolds differ topologically. Removing all physics features increases RMSE by 9.9%, while removing only SASA (cavity energy prior) or only charges (electrostatic prior) causes smaller degradations (4.1% and 2.5% respectively).

Table 10 demonstrates robustness to hyperparameter choices within reasonable ranges. The thermodynamic consistency term ($\:{\lambda\:}_{thermo}$) is most sensitive: too weak (0.1) allows substantial violations, too strong (1.0) overregularizes and degrades accuracy. The sweet spot (0.5) balances physics enforcement with data fitting. Energy conservation weight ($\:{\lambda\:}_{conserv}$= 0.3) is less sensitive, with performance stable across 0.1–0.5. Monotonicity and bounds constraints have smaller effects ($\:{\lambda\:}_{monoton}$ = 0.1, $\:{\lambda\:}_{bounds}$ = 0.2) but still contribute measurably. The selected hyperparameters {0.5, 0.3, 0.1, 0.2} provide optimal trade-off between accuracy and physical consistency across all evaluation metrics.

**Table 10:** Sensitivity to physics loss weights.

$\:{\boldsymbol{\lambda\:}}_{\boldsymbol{t}\boldsymbol{h}\boldsymbol{e}\boldsymbol{r}\boldsymbol{m}\boldsymbol{o}}$	$\:{\boldsymbol{\lambda\:}}_{\boldsymbol{c}\boldsymbol{o}\boldsymbol{n}\boldsymbol{s}\boldsymbol{e}\boldsymbol{r}\boldsymbol{v}}$	$\:{\boldsymbol{\lambda\:}}_{\boldsymbol{m}\boldsymbol{o}\boldsymbol{n}\boldsymbol{o}\boldsymbol{t}\boldsymbol{o}\boldsymbol{n}}$	$\:{\boldsymbol{\lambda\:}}_{\boldsymbol{b}\boldsymbol{o}\boldsymbol{u}\boldsymbol{n}\boldsymbol{d}\boldsymbol{s}}$	RMSE	TVR	Scaffold RMSE	Notes
0.1	0.3	0.1	0.2	0.398	12.1%	0.521	Too weak
0.3	0.3	0.1	0.2	0.378	5.9%	0.467	Better
**0.5**	**0.3**	**0.1**	**0.2**	**0.362**	**3.7%**	**0.428**	**Optimal**
0.7	0.3	0.1	0.2	0.371	2.8%	0.442	Overregularized
1.0	0.3	0.1	0.2	0.389	2.1%	0.478	Much too strong
0.5	0.1	0.1	0.2	0.378	8.4%	0.451	Conservation weak
0.5	0.5	0.1	0.2	0.365	3.2%	0.433	Conservation strong
0.5	0.3	0.0	0.2	0.368	4.1%	0.436	No monotonicity
0.5	0.3	0.3	0.2	0.364	3.5%	0.430	Monotonicity strong
0.5	0.3	0.1	0.0	0.373	5.2%	0.447	No bounds
0.5	0.3	0.1	0.5	0.367	2.9%	0.434	Bounds strong

Bold row: Selected hyperparameters $\:{\lambda\:}_{thermo}$: Gibbs relation weight, $\:{\lambda\:}_{conserv}$: Energy conservation weight $\:{\lambda\:}_{monoton}$: Monotonicity constraint weight, $\:{\lambda\:}_{bounds}$: Physical bounds weight.

### External validation on independent dataset

To assess transferability we evaluate on CombiSolu-Exp^63^ (Table 11), a recently published dataset of 5260 molecules measured using standardized protocols. This dataset has minimal overlap with AqSolDB (< 3% common molecules), providing rigorous assessment of generalization to unseen data from different laboratories.

**Table 11:** External validation on CombiSolu-Exp dataset (*n* = 5260, no overlap with training).

Method	RMSE (↓)	MAE (↓)	*R*²	Pearson *r*	TVR (↓)	Notes
XGBoost^3^	0.834	0.623	0.589	0.768	–	Trained on AqSolDB
GAT^35^	0.621	0.467	0.731	0.855	34.2%	Trained on AqSolDB
ChemBERTa-2^49^	0.548	0.402	0.793	0.891	26.8%	+ 77 M pretrain
Multi-task^31^	0.512	0.378	0.821	0.906	21.4%	Trained on AqSolDB
**PINN (Ours)**	**0.447**	**0.321**	**0.861**	**0.928**	**4.9%**	Trained on AqSolDB
**vs. best baseline**	**18%**	**15%**	**+ 4.8%**	**+ 2.2%**	**77%**	Relative improvement

Table 11 confirms that physics-informed learning transfers successfully to independent data. Our model achieves RMSE 0.447 on CombiSolu-Exp despite never seeing these molecules during training, outperforming ChemBERTa-2 (0.548 RMSE) by 18%. The performance gap is slightly larger than on AqSolDB test set (18% vs. 14%), suggesting physics constraints provide particular benefit for cross-dataset generalization. Critically, thermodynamic violation rate remains low (4.9%) even on external data, compared to 26.8% for ChemBERTa-2, demonstrating that learned physical relationships transfer reliably. The modest performance degradation from AqSolDB test (0.362 RMSE) to CombiSolu-Exp (0.447 RMSE) reflects genuine distribution shift between datasets: CombiSolu-Exp includes more exotic molecules (e.g., ionic liquids, surfactants) and uses different experimental protocols (shake-flask vs. potentiometric). However, this 23% degradation is much smaller than for baselines (GAT: 28%, ChemBERTa-2: 30%), confirming superior transferability.

### Computational efficiency

Beyond accuracy, practical deployment requires reasonable computational cost. We analyze training time, inference speed, and scalability.

**Training efficiency.** Our model trains in ~ 2 h on single NVIDIA A100 (40GB) for 200 epochs over AqSolDB (~ 10 K molecules). This is comparable to standard GNN baselines (GAT: 1.8 h, MPNN: 2.1 h) despite additional physics loss terms. The two-stage training procedure (50 epochs warm-up, 150 epochs with physics) adds minimal overhead compared to standard end-to-end training. QM dataset generation is a one- time cost (~ 4500 CPU-hours for 1500 molecules), amortized across all subsequent experiments.

**Inference speed.** At test time, PINN processes ~ 500 molecules/second on CPU (AMD EPYC 7742), enabling high-throughput virtual screening. This is 3× slower than lightweight GNNs (~ 1500 molecules/second) due to multi-task prediction heads, but 10× faster than ChemBERTa-2 (~ 50 molecules/second) due to smaller model size. For GPU inference (A100), throughput increases to ~ 8000 molecules/second, sufficient for screening million-molecule libraries in minutes.

**Scalability.** Model parameters (2.4 M) are modest compared to transfer learning approaches (ChemBERTa-2: 86 M, Uni-Mol: 76 M), enabling deployment on edge devices or embedding in molecular design workflows. Physics constraints scale linearly with batch size and do not add significant memory overhead.

## Discussion

### Key findings and implications

This work establishes physics-informed neural networks as a powerful paradigm for molecular property prediction. Our key findings can be summarized across four dimensions:

**Accuracy.** Incorporating thermodynamic constraints improves predictive performance by 14% (RMSE 0.362 vs. 0.421) compared to state-of-the-art baselines on standard benchmarks. This improvement comes despite using 36× fewer parameters than pretrained transformers, demonstrating that domain knowledge can substitute for massive pretraining.

**Physical consistency.** 96.3% of predictions satisfy fundamental thermodynamic principles, compared to 67.4% for standard neural networks. This dramatic improvement in physical plausibility is crucial for scientific applications where trustworthiness matters more than nominal accuracy.

**Data efficiency.** Physics constraints enable 10× reduction in training data requirements: our model with 10% data matches baseline performance with full dataset. This advantage is transformative for data-scarce domains (novel therapeutic modalities, emerging materials, toxicology) where experimental measurements are expensive or time-consuming.

**Extrapolation.** Performance degradation on novel chemical scaffolds is only 18% (RMSE 0.362 → 0.428), compared to 57% for baselines (0.421 → 0.659). This reliable extrapolation is essential for drug discovery, where predictions are most valuable for unexplored chemical space.

Beyond these quantitative improvements, our approach offers several qualitative advantages. First, multi-task predictions expose learned physics (energy components), enabling mechanistic interpretation of solubility trends. Second, physics-aware uncertainty quantification provides actionable confidence estimates, flagging predictions likely to fail due to thermodynamic inconsistency. Third, the framework is modular and extensible, allowing incorporation of additional physical constraints (e.g., pH-dependent ionization, temperature effects, co-solvent mixtures) without architectural changes.

### When does physics-informed learning help most?

Analysis of performance stratified by molecular properties (Table 6) reveals when physics constraints provide greatest benefit:

**Large molecules (MW > 500 Da).** Physics-informed learning shows 35% improvement for large molecules, where cavity formation energy dominates thermodynamics. The architectural constraint $\:{\varDelta\:G}_{cavity}\:\approx\:\:\gamma\:\cdot SASA$ provides strong inductive bias, preventing spurious correlations between size and solubility that data-driven models learn.

**Extreme lipophilicity (**$\:\boldsymbol{L}\boldsymbol{o}\boldsymbol{g}\boldsymbol{P}$ > 5). For highly lipophilic compounds, 35% improvement reflects correct physical relationship between hydrophobicity and solvation. Monotonicity constraint (solubility decreases with $\:LogP$) guides predictions even when training data is sparse in this regime.

**High polarity (TPSA > 150 Ų).** For polar molecules, 33% improvement demonstrates accurate modeling of electrostatic interactions. Explicit prediction of $\:{\varDelta\:G}_{electrostatic}$ helps disentangle effects of charge, shape, and hydrogen bonding.

**Novel Scaffolds.** Scaffold split shows 35% improvement, indicating physics constraints enable reliable extrapolation to unseen chemotypes. This is consistent with PINN literature: embedding domain knowledge provides inductive bias for generalization beyond training distribution.

**Low-data regime.** With 1% training data, 28% improvement grows to 21% at 10% data, then stabilizes at 14% with full dataset. This pattern suggests physics constraints act as powerful regularizers in data-scarce regime, with benefits persisting even when data is abundant. Conversely, physics-informed learning provides minimal benefit for:

**Simple molecules (MW < 200 Da).** For small molecules with few functional groups, standard GNNs perform well (~ 0.35 RMSE) without physics, leaving little room for improvement. However, PINN still maintains slight advantage (0.31 vs. 0.35) due to better calibrated uncertainty.

**Well-represented chemical space.** For molecules similar to training data (Tanimoto > 0.7), even baseline models generalize reliably. Physics constraints provide 5–8% improvement, smaller than for novel scaffolds but still meaningful.

**Very large datasets.** With > 50 K training examples, standard deep learning approaches asymptote toward optimal performance, reducing gains from physics regularization. However, computational cost of generating such datasets often exceeds cost of physics-informed modeling. These trends suggest physics-informed learning is most valuable when: (1) training data is limited or expensive, (2) extrapolation to novel chemical space is required, (3) physical plausibility is crucial for trust and interpretability, or (4) edge cases (large molecules, extreme properties) are common in application domain.

### Comparison to related approaches

Our work builds on and extends several research directions:

**Physics-informed neural networks**. Traditional PINNs^18–22,42,43^ enforce differential equations (PDEs, ODEs) arising from continuum mechanics or field theories. We adapt this framework to molecular-scale phenomena, where physics is encoded through thermodynamic relationships rather than differential equations. Our constraints (Gibbs relation, energy conservation, monotonicity) are differentiable algebraic expressions rather than PDE residuals, enabling seamless integration into molecular property prediction.

**Multi-task learning**. Prior work^51,64–66^ shows predicting multiple properties jointly can improve accuracy through shared representations. However, these approaches lack explicit physical constraints linking tasks. We extend multi-task learning by enforcing thermodynamic relationships between outputs ($\:{{\varDelta\:}{G}}_{{t}{o}{t}{a}{l}}\:=\:{\varSigma\:}\:{{\varDelta\:}{G}}_{{i}},\:{\varDelta\:}{G}=\:-{R}{T}\:{l}{n}\:{S}$), ensuring consistency rather than merely correlation.

**Transfer learning.** Large-scale pretraining (ChemBERTa-2^40^, Uni-Mol^49^ leverages unlabeled molecules to learn transferable representations. While effective, these models still violate physics constraints and struggle with extrapolation. Our approach appears complementary: we hypothesize that physics-informed fine-tuning could potentially further improve pretrained models by adding thermodynamic consistency. Testing this hypothesis—training our physics-informed objective on top of ChemBERTa-2 or Uni-Mol representations—represents an exciting direction for future work that could potentially combine the benefits of large-scale pretraining and explicit domain knowledge. Our results suggest these paradigms are synergistic rather than competing: physics constraints with 10 K training examples achieve comparable performance to pretraining on 77 M molecules, indicating that explicit domain knowledge can dramatically improve data efficiency. Combining both—pretraining for general molecular representations plus physics-informed fine-tuning for task-specific constraints—represents a promising direction for future research.

**Quantum chemistry integration.** Some studies^26,47,48^ incorporate DFT calculations directly into neural network training (e.g., SchNet, DimeNet++). These architectures predict atomic forces or energies by learning from quantum chemistry, but require 3D coordinates and are computationally expensive. We use DFT data more efficiently: QM-derived energy components serve as auxiliary supervision for only 15% of training set (1500 of 10,000 molecules), providing ground truth without constraining architecture.

**Mechanistic modeling.** Traditional cheminformatics^1–3^ uses mechanistic models (COSMO-RS, SMD) that solve thermodynamic equations explicitly. While physically rigorous, these approaches require expensive QM calculations per molecule (minutes to hours) and struggle with complex solvents or mixtures. Our neural network approximates mechanistic calculations efficiently (~ 2 ms per molecule) while maintaining physical consistency through constraints.

Our approach uniquely combines benefits of multiple paradigms: speed of neural networks, rigor of mechanistic models, data efficiency of physics-informed learning, and interpretability of multi-task architectures. This synthesis enables applications requiring both accuracy and trustworthiness.

**Future directions**

**Combining pretraining and physics constraints**

An important untested hypothesis is whether physics-informed learning can enhance pretrained models. Our results demonstrate that explicit thermodynamic constraints with 10 K training examples achieve comparable performance to pretraining on 77 M molecules (ChemBERTa-2), suggesting these paradigms provide complementary benefits. A natural next step would be applying our physics-informed fine-tuning protocol to pretrained models: initializing from ChemBERTa-2 or Uni-Mol representations, then training with our physics-constrained objective. This could potentially achieve superior performance by combining general molecular representations from pretraining with task-specific thermodynamic constraints. We leave this promising direction for future investigation.

### Limitations and failure modes

Despite strong overall performance, our approach has several limitations worth acknowledging:

**Simplified thermodynamics.** We decompose solvation energy into four components (cavity, electrostatic, vdW, Hbond), neglecting higher-order terms (e.g., polarization, charge transfer, specific ion effects). For most organic molecules in water, this approximation is reasonable (residuals < 10% of total energy). However, for ionic liquids, metal complexes, or exotic solvents, additional physics may be necessary.

**Hydrogen bonding approximation.** Our semi-empirical H-bond model ($\:{{\varDelta\:}{G}}_{{H}{b}{o}{n}{d}}\:=\:-{{N}}_{{d}{o}{n}{o}{r}}\cdot {{w}}_{{d}{o}{n}{o}{r}}\:-\:{{N}}_{{a}{c}{c}{e}{p}{t}{o}{r}}\cdot {{w}}_{{a}{c}{c}{e}{p}{t}{o}{r}}$) neglects geometric details such as bond angles, distances, and cooperative effects. While learned weights match literature values on average, individual H-bonds can vary by 2–3 kJ/mol depending on configuration^55^. A more sophisticated geometric treatment could improve accuracy at increased complexity cost. However, for physics-informed supervision, the current approach provides effective inductive bias. Validation against NBO analysis for 100 molecules (R²=0.84, MAE = 2.8 kJ/mol) confirms acceptable accuracy. The trainable weights allow the network to learn corrections, and high-dimensional GNN/SMILES representations capture contextual information beyond simple donor/acceptor counts.

**Solvent (water).** All experiments focus on aqueous solubility. Extending to organic solvents or solvent mixtures requires solvent-dependent physics constraints (e.g., γ varies with solvent, dielectric constant $\:{{\epsilon\:}}_{{r}}$ differs). The framework generalizes naturally, but each solvent requires recalibration of physics weights and potentially new constraints.

**Temperature dependence.** We predict solubility at 25 °C only. Temperature-dependent solubility prediction requires additional physics (entropy vs. enthalpy contributions, temperature-dependent dielectric) and experimental data spanning temperature ranges. This is feasible within our framework but left for future work.

**Tautomers and ionization.** We use canonicalized neutral forms, ignoring tautomerization and ionization equilibria. For ionizable compounds (pKa near neutral pH), predicted solubility may be inaccurate. Incorporating pH-dependent ionization requires multi-species equilibrium model, adding complexity.

**Failure cases.** Examining predictions with largest errors reveals common failure modes:

- Zwitterions (amino acids, betaines): Simultaneous positive and negative charges violate our neutral molecule assumption.
- Salts and complexes: Multi-component stoichiometry not captured by single-molecule model.
- Aggregating molecules (surfactants): Micellization changes effective solubility.
- Reactive compounds: Decomposition or tautomerization in solution.

These difficult chemistry categories comprise 3.1% of the test set (47 of 1497 molecules). Importantly, there is strong overlap (76%, 42 of 55 molecules) with the 3.7% exhibiting major thermodynamic violations (TVR > 5 kJ/mol) reported in Sect.  4.2. This correspondence is scientifically meaningful: difficult chemistry causally leads to physics violations in most cases, validating our physics-aware uncertainty quantification as an effective diagnostic tool. The remaining 24% of violations (13 molecules) occur in apparently ‘normal’ chemical structures, likely reflecting training data gaps or subtle structural features not captured by simple categorization. Conversely, 11% of difficult-chemistry molecules (5 of 47) show no major violations, demonstrating the framework sometimes succeeds even for challenging cases.

### Broader impact and future directions

**Immediate applications.** Physics-informed molecular property prediction has near-term applications in:

- **Drug discovery**: Solubility is a key ADMET property; our model enables high-throughput screening with reliable uncertainty estimates.
- **Formulation science**: Predicting solubility in mixed solvents or with excipients.
- **Environmental risk assessment**: Estimating bioaccumulation and aquatic toxicity (both depend on solubility).
- **Materials discovery**: Designing functional molecules (OPV, OLED) with target solubility.

**Extensions to other properties.** The PINN framework generalizes to other molecular properties with known physics:

- LogP (octanol-water partition): Similar thermodynamic decomposition, different solvent.
- pKa: Acid-base equilibrium, related to $\:{\varDelta\:G}_{deprotonation}$.
- Permeability: Lipinski’s rule, membrane partitioning physics.
- Binding affinity: Protein-ligand thermodynamics, though more complex.

Each property requires domain-specific constraints but follows the same template: encode known physics as differentiable loss terms.

**Active learning and uncertainty-guided design.** Physics-aware uncertainty enables active learning: prioritize experimental validation for high-uncertainty compounds flagged by thermodynamic violations. This closes the loop between prediction and measurement, iteratively improving models while minimizing experimental cost.

**Interpretable AI for science.** Multi-task predictions expose learned physics (energy components), enabling mechanistic interpretation. Chemists can inspect why a molecule is predicted to be soluble (e.g., strong H- bonding overcomes large cavity penalty), facilitating rational design. This interpretability is crucial for scientific applications where understanding matters as much as prediction.

**Challenges and open questions.** Several challenges remain:

- Optimal balance between data-driven flexibility and physics-imposed rigidity: Too much physics constraint may prevent learning genuine but unexpected patterns.
- Handling domain shift: How to adapt physics constraints when training and test distributions differ substantially.
- Uncertainty quantification: Current approach combines dropout and physics violations; more principled Bayesian treatment may improve calibration.
- Scaling to complex multi-component systems (mixtures, proteins, crystals): Thermodynamic complexity grows rapidly.

Addressing these challenges will require interdisciplinary collaboration between machine learning researchers and domain scientists. The success of physics-informed learning depends critically on encoding correct domain knowledge—garbage constraints produce garbage predictions.

## Conclusion

We have introduced the first physics-informed neural network framework for molecular solubility prediction, demonstrating that incorporating thermodynamic constraints dramatically improves accuracy, physical consistency, data efficiency, and extrapolation capability. Our approach achieves 14% improvement in predictive accuracy (RMSE 0.362 vs. 0.421), 88% reduction in physics violations (3.7% vs. 32.6%), 10× data efficiency (10% data matches baseline with full data), and 35% better extrapolation to novel chemical scaffolds compared to state-of-the-art purely data-driven methods.

The key innovation is encoding thermodynamic solvation theory—Gibbs free energy decomposition, energy conservation, monotonicity relationships—as differentiable constraints in the learning objective. This ensures predictions satisfy fundamental physical principles while maintaining the flexibility of deep learning. Multi-task architecture simultaneously predicts solubility and its thermodynamic components (cavity formation, electrostatic interactions, van der Waals forces, hydrogen bonding), with learned representations validated against quantum chemistry ground truth. Physics-aware uncertainty quantification provides reliable confidence estimates, enabling deployment in safety-critical applications.

Failure mode analysis reveals that molecules with major thermodynamic violations (3.7% of predictions) are strongly enriched for chemical classes known to challenge continuum solvation models (charged species, macrocycles, polyaromatics), with 76% overlap between physics violations and ‘difficult chemistry’ categories. This correspondence validates physics-aware uncertainty quantification as an effective diagnostic tool for identifying problematic predictions.

Beyond solubility, this work establishes a general methodology for incorporating domain knowledge into molecular property prediction. The framework is modular and extensible: any property with known physics can benefit from analogous treatment. As machine learning increasingly penetrates scientific domains, physics- informed approaches offer a promising path toward models that are accurate, trustworthy, and interpretable— combining the flexibility of data-driven learning with the rigor of mechanistic understanding. Future work should explore broader applications (other solvents, temperature dependence, mixture effects), develop principled approaches for balancing physics constraints against data-driven flexibility, and investigating whether physics-informed fine-tuning can enhance large pretrained models (ChemBERTa-2, Uni-Mol) by adding thermodynamic consistency constraints to learned representations.

The results demonstrate that physics and machine learning are complementary rather than competing paradigms. Neural networks excel at approximating complex nonlinear relationships from data, while physics provides inductive bias for extrapolation and interpretability. By thoughtfully combining these strengths, we can build models that are both powerful and principled—advancing not just predictive accuracy, but scientific understanding.

## Supplementary Information

Below is the link to the electronic supplementary material.

Supplementary Material 1

Supplementary Material 2

## Data Availability

The AqSolDB dataset is publicly available at https://www.kaggle.com/datasets/sorkun/aqsoldb-a-curated-aqueous-solubility-dataset. Codes would be available through corresponding author.

## Code availability

All simulation code has been attached as supplementary material.
