# Supplementary material for: Physics-informed deep learning for molecular solubility prediction: integrating thermodynamic constraints with neural network architectures
Source: Sci Rep. 2026 Apr 27;16:19450. doi: 10.1038/s41598-026-49635-4 (PMC13287665; doi:10.1038/s41598-026-49635-4)
Supplement: Supplementary file 1 — Supplementary Material 1 [file 41598_2026_49635_MOESM1_ESM.docx]

**All Tables for Manuscript Revision**

*Complete Tables with Standard Formatting
Text Before and After Each Table*

This document contains all new tables required for the manuscript completion. Each table includes: (1) Text to insert before table, (2) The formatted table, (3) Table caption/note, (4) Text to insert after table.

**Table S1: Sensitivity Analysis - Decomposition Protocol Robustness**

**BEFORE TABLE S1:**

To validate that our neural network learns genuine thermodynamic relationships rather than merely reproducing empirical decomposition formulas, we conducted comprehensive sensitivity analysis. We systematically varied: (1) the cavity surface tension coefficient γ by ±20%; (2) hydrogen bonding estimation formulas using three alternative approaches; (3) training with complete removal of deterministic components (cavity or H-bond supervision); and (4) alternative solvation models (PCM instead of SMD). Results are summarized in Table S1.

**TABLE S1:**

| Protocol Variation | Test RMSE | ${\Delta G}_{cav}$ R² | ${\Delta G}_{elec}$R² | ${\Delta G}_{vdW}$R² | ${\Delta G}_{Hbond}$ R² | TVR (%) |
| --- | --- | --- | --- | --- | --- | --- |
| Baseline (γ=0.0227) | 0.362 | 0.914 | 0.852 | 0.786 | 0.881 | 3.7 |
| Low γ (0.018, -20%) | 0.368 | 0.908 | 0.851 | 0.783 | 0.879 | 4.1 |
| High γ (0.027, +20%) | 0.357 | 0.919 | 0.853 | 0.789 | 0.883 | 3.4 |
| Equal H-bond weights | 0.371 | 0.914 | 0.849 | 0.785 | 0.867 | 4.3 |
| Geometric H-bonds | 0.359 | 0.914 | 0.852 | 0.787 | 0.893 | 3.5 |
| No cavity supervision | 0.389 | 0.782 | 0.851 | 0.781 | 0.881 | 5.9 |
| No H-bond supervision | 0.378 | 0.914 | 0.850 | 0.784 | 0.623 | 4.8 |
| PCM decomposition | 0.374 | 0.891 | 0.839 | 0.771 | 0.856 | 4.5 |

***Table S1.*** *Sensitivity analysis: Model performance under alternative energy decomposition protocols. Test RMSE in log units on AqSolDB test set (n=1,497). TVR = Thermodynamic Violation Rate (|*${\Delta G}_{pred} - {\Delta G}_{components}$*| > 5 kJ/mol). All RMSE differences >2% are statistically significant (p < 0.01, paired t-test). Results demonstrate model robustness: performance varies <10% across all decomposition alternatives, indicating the neural network learns genuine thermodynamic relationships rather than memorizing empirical formulas. The baseline row uses Pimentel-McClellan H-bond formula and γ=0.0227 kJ/(mol·Å²) for cavity energy. Alternative H-bond approaches include equal weighting (5.0 kJ/mol for both donors and acceptors) and geometry-dependent estimates incorporating bond angles. PCM (Polarizable Continuum Model) provides an alternative to SMD for solvation energy decomposition.*

**TEXT AFTER TABLE S1:**

As shown in Table S1, varying the cavity coefficient γ by ±20% results in only ±3% RMSE variation (0.357-0.368), demonstrating robustness to parameter uncertainty. Similarly, alternative H-bond formulas cause <3% performance variation (0.359-0.371 RMSE). Removing cavity supervision entirely increases RMSE by 7.5% (0.362→0.389), with cavity prediction R² dropping from 0.914 to 0.782—a modest degradation that confirms the GNN encoder learns implicit surface area representations even without explicit supervision. Removing H-bond supervision increases RMSE by 4.4%, while using an alternative solvation model (PCM) increases RMSE by 3.3%. The maximum performance variation across all eight protocol alternatives is ±7%, well below typical model uncertainty, confirming that learned representations are stable and physics-based rather than artifacts of specific decomposition choices. This robustness arises because: (1) the neural network has sufficient capacity (~2.4M parameters) to learn corrections beyond initial empirical estimates; (2) physics constraints (energy conservation, monotonicity) provide independent supervision; and (3) experimental solubility provides ultimate validation regardless of intermediate approximations.

**Table S2: H-bonding Prediction Head Architecture Comparison**

**TEXT BEFORE TABLE S2:**

To justify our semi-constrained H-bonding head design (linear form with learned weights), we compared against unconstrained architectures that could potentially capture geometric and cooperative effects ignored by simple donor/acceptor counting. We trained four alternative architectures using identical protocols and evaluated on the same test set. Results are presented in Table S2.

**TABLE S2:**

| Architecture | Test RMSE | H-bond R² | Parameters | Learned Weights | Interpretability |
| --- | --- | --- | --- | --- | --- |
| Semi-constrained linear (baseline) | 0.362 | 0.881 | 2 | $w_{d}$=6.31, $w_{a}=$4.18 | High |
| Unconstrained 3-layer MLP | 0.359 | 0.893 | ~75,000 | N/A | Low |
| Attention over H-bond pairs | 0.361 | 0.887 | ~120,000 | N/A | Medium |
| No H-bonding component | 0.378 | N/A | 0 | N/A | N/A |

***Table S2.*** *Comparison of H-bonding prediction head architectures. Test RMSE in log units on AqSolDB test set (n=1,497). H-bond R² evaluated on QM-Thermo subset (n=1,500) with ground-truth H-bond energies from quantum calculations. Learned weights in kJ/mol (*$w_{d}$ *= donor weight,* $w_{a}$ *= acceptor weight) agree well with literature values (6-7 kJ/mol for donors, 4-5 kJ/mol for acceptors). The semi-constrained linear design achieves 99.2% of the best performance (0.362 vs 0.359 for unconstrained MLP) using only 0.003% of the parameters (2 vs ~75,000). This demonstrates optimal balance between predictive accuracy, sample efficiency (only 1,500 molecules have H-bond supervision), and scientific interpretability. The unconstrained architectures provide marginal improvements (0.8-0.3% RMSE reduction) at the cost of substantial complexity and loss of interpretable learned weights. Geometric and cooperative effects are implicitly captured in the 576-dimensional combined representation from GNN/SMILES encoders that feeds into this head.*

**TEXT AFTER TABLE S2:**

Table S2 demonstrates that our semi-constrained architecture provides an optimal cost-benefit trade-off. The unconstrained 3-layer MLP achieves the best H-bond prediction accuracy (R²=0.893), but requires 37,500 times more parameters for only 0.8% RMSE improvement. Given that only 1,500 molecules in our training set have explicit H-bond energy labels from quantum calculations, the unconstrained MLP risks overfitting these limited supervision signals. The attention mechanism over H-bond pairs, while theoretically appealing for capturing pairwise interactions, provides no practical benefit (0.3% improvement) and increases parameters by 60,000×. Importantly, the semi-constrained design yields interpretable learned weights ($w_{donor}$=6.31, $w_{acceptor}$=4.18 kJ/mol) that closely match literature values, enabling scientific validation. Removing the H-bonding component entirely (last row) degrades overall RMSE by 4.4%, confirming that H-bond supervision provides meaningful inductive bias. We therefore retain the semi-constrained linear architecture as it balances predictive accuracy, parameter efficiency, sample efficiency, and scientific interpretability—critical considerations for physics-informed learning with limited labeled data.

**Table S3: Diagnostic Analysis of Cavity Energy Learning**

**TEXT BEFORE TABLE S3:**

The high cavity energy correlation (R²=0.914, Table 3 main text) raises the question of whether the model merely performs trivial regression of SASA to cavity energy via the deterministic relationship ${\Delta G}_{cav} \approx\gamma\times SASA$. To investigate this, we conducted three diagnostic analyses examining prediction residuals, out-of-distribution extrapolation, and ablated model performance. Results are summarized in Table S3.

**TABLE S3:**

| Analysis | Metric | Value | Interpretation |
| --- | --- | --- | --- |
| Theoretical scaling | SASA vs $\Delta G\_cav,theory$ | R² = 1.000 | **By definition** |
| Model prediction | SASA vs $\Delta G\_cav,pred$ | R² = 0.891 | **Less than perfect indicates corrections** |
| Theory vs prediction | $\Delta G\_cav,theory vs \Delta G\_cav,pred$ | R² = 0.914 | **Model learns beyond simple scaling** |
| Residual Correlations |  |  |  |
| Asphericity | Shape vs residuals | R² = 0.31 | **Shape corrections learned** |
| Surface roughness | Topology vs residuals | R² = 0.28 | **Topology corrections learned** |
| Buried surface | Burial vs residuals | R² = 0.24 | **Occlusion effects learned** |
| Out-of-distribution (SASA > 95th percentile) |  |  |  |
| Simple linear | OOD MAE | 8.2 kJ/mol | **Poor extrapolation** |
| Our model | OOD MAE | 4.7 kJ/mol | **43% better (nonlinear learning)** |

***Table S3.*** *Diagnostic analysis of cavity energy learning. Residuals defined as* $\varepsilon= \Delta G\_cav,pred - 0.0227\times SASA$*, representing deviations from simple linear scaling. Shape descriptors (asphericity, surface roughness, buried surface fraction) computed using RDKit molecular descriptors. Out-of-distribution (OOD) test includes 75 molecules from external validation set with SASA > 500 Ų (95th percentile of training data). Simple linear model uses* $\Delta G\_cav = 0.0227\times SASA$ *with no learned corrections. Results demonstrate genuine physical learning beyond trivial SASA regression: (1) Residuals correlate with molecular shape/topology features not directly encoded in SASA, indicating learned corrections for geometric effects. (2) Out-of-distribution extrapolation substantially outperforms simple linear scaling, confirming learned nonlinear relationships. (3) The discrepancy between SASA vs theory (R²=1.0 by definition) and SASA vs predictions (R²=0.891) indicates the model has learned to deviate from perfect linear scaling when physically appropriate.*

**TEXT AFTER TABLE S3:**

Table S3 provides multiple lines of evidence that the model learns genuine physics beyond trivial SASA regression. First, the residual analysis (rows 5-7) shows that deviations from simple linear scaling ($\varepsilon= \Delta G\_cav,pred - 0.0227\times SASA$) correlate significantly with molecular shape descriptors: asphericity (R²=0.31), surface roughness (R²=0.28), and buried surface fraction (R²=0.24). These correlations indicate the model has learned that non-spherical molecules, molecules with irregular surface topology, and molecules with significant buried surface area all require cavity formation energies that deviate from simple SASA scaling—consistent with solvent reorganization theory. Second, the out-of-distribution analysis (rows 9-10) demonstrates that when tested on molecules with SASA values exceeding the 95th percentile of training data (SASA > 500 Ų), our model's extrapolation error (4.7 kJ/mol) is 43% lower than simple linear scaling (8.2 kJ/mol). This confirms the model has learned nonlinear corrections that improve predictions even outside the training distribution. Third, comparing rows 1-3, we observe that SASA vs theory has perfect correlation (R²=1.0 by definition), but SASA vs predictions has lower correlation (R²=0.891), while theory vs predictions maintain high correlation (R²=0.914). This pattern indicates the model has learned systematic deviations from perfect linear scaling. Combined with the ablation result from Table 9 (removing SASA features drops cavity R² from 0.914 to 0.881—only 3.6%), these results confirm that while SASA provides valuable inductive bias, the model learns substantial corrections for molecular geometry and topology, representing genuine physical understanding rather than trivial feature regression.

**Table S4: Comparison of Penalty Functions for Physics Constraints**

**TEXT BEFORE TABLE S4:**

To justify the use of ReLU penalty functions in our physics-informed loss (Equations 10-13) despite non-differentiability at x=0, we conducted systematic comparison against four smooth alternative penalty functions. All configurations used identical architectures, training protocols, and hyperparameters, differing only in the penalty function applied to constraint violations. Five random seeds were trained for each configuration to assess statistical significance. Results are summarized in Table S4.

**TABLE S4:**

| Penalty Function | Test RMSE | TVR (%) | Training Time (h) | Gradient Norm Std | Convergence Epoch |
| --- | --- | --- | --- | --- | --- |
| ReLU (baseline) | 0.362 | 3.7 | 2.0 | 0.021 | 180 |
| Softplus (β=10) | 0.374 | 5.2 | 2.1 | 0.019 | 185 |
| Quadratic (L2) | 0.368 | 4.1 | 2.2 | 0.034 | 195 |
| ELU (α=1.0) | 0.365 | 3.9 | 2.0 | 0.024 | 183 |
| Huber (δ=1.0) | 0.365 | 3.9 | 2.1 | 0.022 | 182 |

***Table S4.*** *Comparison of penalty functions for enforcing physics constraints. Test RMSE in log units on AqSolDB test set (n=1,497). TVR = Thermodynamic Violation Rate (|*$\Delta G\_total - \Delta G\_components$*| > 5 kJ/mol). Training time measured on single NVIDIA A100 GPU. Gradient norm std represents standard deviation of loss gradient magnitudes during training epochs 100-200 (after initial transient). Convergence epoch is when validation loss plateau is reached (no improvement >0.001 for 20 consecutive epochs). Penalty functions: ReLU(x) = max(0,x); Softplus(x) = (1/β)log(1+exp(βx)) with β=10 approximating ReLU; Quadratic(x) = x² for x>0; ELU(x) = x for x>0, α(exp(x)-1) for x≤0 with α=1.0; Huber(x) = x²/2 for |x|≤δ, δ(|x|-δ/2) for |x|>δ with δ=1.0. Statistical significance: Paired t-test comparing ReLU vs each alternative shows p<0.05 for softplus and quadratic, p>0.1 for ELU and Huber (5 random seeds each). All configurations exhibit stable gradient flow with standard deviations <0.04, indicating no practical training difficulties from ReLU's non-differentiability.*

Table S4 demonstrates that ReLU provides the optimal balance of predictive accuracy, constraint enforcement, and computational efficiency. ReLU achieves the best test RMSE (0.362) and lowest TVR (3.7%), indicating strongest physics constraint satisfaction. The smooth alternative softplus performs notably worse (RMSE 0.374, +3.3%; TVR 5.2%, +40% violations) because its gentle transition around zero allows more boundary violations—the softness that ensures differentiability everywhere simultaneously permits gradual constraint relaxation during training. Quadratic penalties over-penalize small violations (RMSE 0.368, +1.7%; TVR 4.1%) because the squared penalty becomes prohibitively large even for minor deviations, forcing the network to prioritize constraint satisfaction over data fitting. ELU and Huber penalties achieve performance nearly matching ReLU (within 1% RMSE, within 5% TVR), demonstrating that smooth approximations can work well—however, they provide no practical advantage over ReLU to justify their added complexity.

Critically, training stability is comparable across all penalty functions. Gradient norm standard deviations range from 0.019 to 0.034, all well within normal ranges for deep learning, with no evidence of instability, explosions, or vanishing. ReLU's gradient norm std (0.021) is actually among the lowest, contradicting any concern about non-differentiability causing optimization difficulties. Convergence epochs are similar (180-195), and training times differ by at most 10%, confirming that ReLU's non-differentiability poses no practical computational burden—modern automatic differentiation frameworks handle subgradients seamlessly, and the proportion of training samples at exactly x=0 (the non-differentiable point) is negligible (<0.1% throughout training).

We therefore adopt ReLU as our primary penalty function, justified by: (1) Best empirical performance (lowest RMSE and TVR); (2) Computational efficiency (fastest forward/backward pass); (3) Training stability comparable to smooth alternatives; (4) Clear physical interpretation (asymmetric penalty aligns with constraint semantics—violations should be penalized, over-satisfaction should not). While smooth alternatives like Huber could be substituted for applications requiring guaranteed differentiability everywhere, ReLU provides optimal balance for physics-informed learning and is the standard choice in the PINN literature.
